# Visiting nature is associated with lower socioeconomic inequalities in well-being in Wales

**DOI:** 10.1038/s41598-023-35427-7

**Published:** 2023-06-15

**Authors:** Joanne K. Garrett, Francis M. Rowney, Mathew P. White, Rebecca Lovell, Rich J. Fry, Ashley Akbari, Rebecca Geary, Ronan A. Lyons, Amy Mizen, Mark Nieuwenhuijsen, Chrissie Parker, Jiao Song, Gareth Stratton, Daniel A. Thompson, Alan Watkins, James White, Susan A. Williams, Sarah E. Rodgers, Benedict W. Wheeler

**Affiliations:** 1 European Centre for Environment and Human Health, University of Exeter Medical School, Truro, UK; 2 School of Geography, Earth and Environmental Sciences, University of Plymouth, Plymouth, UK; 3 Cognitive Science HUB, University of Vienna, Vienna, Austria; 4 Department of Population Data Science, Faculty of Medicine, Health and Life Science, Swansea University Medical School, Swansea University, Swansea, UK; 5 Department of Public Health, Policy and Systems, University of Liverpool, Liverpool, UK; 6 ISGlobal, Barcelona, Spain; 7 Universitat Pompeu Fabra (UPF), Barcelona, Spain; 8 CIBER Epidemiología y Salud Pública (CIBERESP), Madrid, Spain; 9 Public Health Wales, Cardiff, UK; 10 Faculty of Science and Engineering, ASTEM Research Centre, Swansea University, Swansea, UK; 11 Centre for Trials Research, School of Medicine, Cardiff University, Cardiff, UK; 12 Natural Resources Wales, Bangor, UK

**Keywords:** Psychology, Public health

## Abstract

Natural environments can promote well-being through multiple mechanisms. Many studies have investigated relationships between residential green/blue space (GBS) and well-being, fewer explore relationships with actual use of GBS. We used a nationally representative survey, the National Survey for Wales, anonymously linked with spatial GBS data to investigate associations of well-being with both residential GBS and time in nature (N = 7631). Both residential GBS and time spent in nature were associated with subjective well-being. Higher green-ness was associated with lower well-being, counter to hypotheses (predicting the Warwick and Edinburgh Mental Well-Being Scale (WEMWBS): Enhanced vegetation index β =  − 1.84, 95% confidence interval (CI) − 3.63, − 0.05) but time spent in nature was associated with higher well-being (four hours a week in nature *vs.* none β = 3.57, 95% CI 3.02, 4.13). There was no clear association between nearest GBS proximity and well-being. In support of the equigenesis theory, time spent in nature was associated with smaller socioeconomic inequalities in well-being. The difference in WEMWBS (possible range 14–70) between those who did and did not live in material deprivation was 7.7 points for those spending no time in nature, and less at 4.5 points for those spending time in nature up to 1 h per week. Facilitating access and making it easier for people to spend time in nature may be one way to reduce socioeconomic inequalities in well-being.

## Introduction

Promoting mental well-being is an important preventative public health strategy^1^ that aims to reduce the risk of mental health disorders^2^. There is a growing body of evidence that exposure and access to green and blue spaces (GBS) is positively associated with mental health and well-being^3–5^. There are many proposed pathways and mechanisms that may explain relationships between GBS exposure and well-being^6–8^. Markevych, et al.^6^ categorises these as: reducing harm from the environment, including the mitigation of urban noise^9–11^; building capacities, such as increased social cohesion ^12,13^ and physical activity^14^; and restoring capacities depleted by everyday life, including cognitive capacities^15–17^, and reducing stress^18–20^. Although some of these benefits can be obtained by living near GBSs (e.g. restoration through visual exposure, noise mitigation), other potential pathways to well-being (e.g. physical activity, social interactions) are assumed to require visiting GBSs. While the evidence on residential exposure and health and well-being has grown rapidly^3^, few studies have explored the relationships between GBS visits and well-being (although see Refs.^20–25^).

The methods used to assess exposure to nature vary widely. Residential exposure has been operationalised in a variety of ways, typically as measures of quantity or accessibility^3,26^ in the area around the home. Measures of the use of GBS also vary^27^. Each of these measures differ slightly in their likely associated pathways to mental health and well-being. Here, we consider both green and blue spaces as well as measures of quantity, accessibility and use, which is comparatively rare.

We used the Secure Anonymised Information Linkage (SAIL) Databank^28–30^ to link respondents to the National Survey of Wales (NSW) with high spatial resolution GBS metrics for the area immediately around their home. Our residential GBS measures include measures of both quantity and accessibility. These were: residential green-ness as measured by the Enhanced Vegetation Index (EVI; quantity); and proximity to the nearest GBS from the home (access). Our measure for actual use of GBS was weekly time spent outdoors for leisure in open spaces such as paths, woodland, parks and farmland (hereafter referred to as ‘time in nature’) which was derived from a number of questions in the NSW.

Mental well-being is thought to be multi-dimensional, incorporating the balance of positive and negative emotions (affect); an evaluation of how one’s life is going (evaluative); and purpose or feeling life is worthwhile (eudaimonic)^31–33^. The Warwick-Edinburgh Mental Well-being Scale (WEMWBS) is a metric intended to capture affective, evaluative and eudaimonic wellbeing dimensions^32^. This is often applied in studies investigating relationships between well-being and GBS^34–39^. We therefore applied this measure of well-being along with a single–item question about life satisfaction (an additional measure of evaluative well-being). This gave a longer-term view of an individual’s mental well-being than WEMWBS, and has also often been used in studies investigating relationships between well-being and GBS^4,21,24,35,37,40^.

Access/proximity to green and blue spaces have been found to mitigate socio-economic inequalities in mental health and well-being, the ‘equigenesis’ hypothesis^41,42^. Here, we extend this work to test whether the use of these spaces may also reduce mental health inequalities. We used a binary measure of material deprivation based on an additive score summarising whether or not the participant could afford a series of items. This measure is designed to capture long-term poverty, rather than short term financial strain^43,44^.

Thus, we considered the following hypotheses: greater residential GBS exposure (hypothesis 1a) and greater GBS use (hypothesis 1b) is associated with higher subjective mental well-being and life satisfaction for Welsh residents. To test the equigenesis theory, our hypothesis 2 was: associations between residential GBS exposure (2a) and GBS use (2b) with subjective well-being and life satisfaction are modified by household deprivation.

## Results

### Participants

There were a total of 19,869 respondents to the National Survey for Wales in 2016/17 and 2018/19 combined^45,46^, of which 1453 could not be linked (n = 18,416). Of these, there were 11,378 respondents who completed the Natural Resources for Wales module of the survey. After excluding cases with missing data for exposures, outcomes or covariates there were 7631 adults in the analysis sample (see “Methods and data”).

#### Descriptive statistics

Residential green-ness as measured by the Enhanced Vegetation Index (EVI) has values theoretically ranging from − 1 to + 1. Nearly 60% (weighted %) of survey respondents in Wales lived in areas with an EVI range of 0.2–0.4, with only 10% with an EVI of < 0.2, and ~ 6% with EVI ≥ 0.6 (Table 1). For context, typical values in broadleaf woodlands range from ~ 0.2–0.3 in winter to ~ 0.6–0.7 in summer ^47^, and the mean EVI of a 500 m buffer around the home of a study in London (UK) was 0.37^48^ (negative values typically indicate a lack of vegetation, or presence of water).

**Table 1:** Sample descriptive statistics and mean well-being scores by GBS exposure categories (full descriptive statistics in Supplementary Table 2.1).

Category	Counts	Weighted %	WEMWBS^a^		Life satisfaction^b^	
			Mean	SD	Mean	SD
Total	7631		50.92	9.36	7.77	1.84
Residential green-ness (EVI)						
0– < 0.2	815	10.02	50.92	9.17	7.71	1.79
0.2– < 0.4	4330	56.26	51.15	9.14	7.82	1.81
0.4– < 0.6	2100	28.22	51.19	9.28	7.93	1.69
0.6– < 0.82	386	5.50	52.46	8.05	7.92	1.61
GBS proximity (m)						
0– < 100	2189	29.53	51.15	9.63	7.85	1.79
100– < 300	3957	51.28	51.19	9.03	7.83	1.76
300– < 500	1197	15.31	51.02	8.78	7.84	1.76
500–1100	288	3.88	52.61	7.79	8.00	1.69
GBS use						
Weekly time in nature (mins)						
0	2309	26.19	48.89	10.25	7.46	2.14
> 0– < 60	1210	16.25	51.30	8.54	7.98	1.62
60– < 120	953	13.51	51.30	8.16	7.89	1.62
120– < 240	1205	17.08	51.68	8.29	7.89	1.59
240–420	1954	26.97	53.06	8.82	8.08	1.55

^a^WEMWBS can range from 14 to 70 (higher scores indicate more positive mental well-being).

^b^0 = ‘not at all satisfied’ and 10 = ‘completely satisfied’ with “your life nowadays”.

In terms of home proximity to the nearest publicly accessible GBS, the majority of people (81%) lived less than 300 m, and only 4% lived 500–1100 m, from the nearest green or blue space. Time spent in nature was measured by self-reported recreational GBS use, and over 40% of the sample reported spending more than two hours in nature per week, although one quarter reported spending no time per week (Table 1). Thirteen percent were classified as living in material deprivation and 60% lived in larger urban areas (Supplementary Table 2.1).

### GAM-based modelling decisions

Given some evidence of non-linear relationships between GBS use and well-being^24^ we took the relatively novel approach of first applying generalised additive models (GAMs; Supplemental materials Sect. 1), to indicate the nature of the underlying relationship (e.g. linear or non-linear). We then used these results to inform generalised linear models (GLMs), for example, categorising values where appropriate. This facilitated the interpretation of any patterns in statistical strength. Full details can be found in Supplementary materials Sect. 1 and modelling decisions are summarised here. GAMs indicated that there was no evidence of association between residential EVI and WEMWBS. Given previous literature explored only urban residents^41,49^, and potential for correlation between urbanity and residential GBS, we stratified by urban residents as a post-hoc analysis. A (negative) linear association was found between residential EVI and WEMWBS for urban residents only. Therefore, residential EVI was modelled with a linear term when predicting both WEMWBS and life satisfaction in subsequent GLMs.

Complex non-linear relationships were found between proximity to the nearest GBS and subjective well-being for categories of urban/rural and deprivation status. As such, categories were derived based on visually inspecting the relationships for both identified complex relationships (Supplemental materials Sect. 1; Figs. 1.6, 1.7) and adjusting cut-off points to the nearest 50 m. These were 0– < 100, 100– < 300, 300– < 500 and 500–1100 m. Categories for proximity to nearest GBS correspond with previous work in the literature^50^ and 300 m is considered to correspond to an approximate walking time of 5 min^51^.

Complex non-linear relationships were found between time in nature and subjective well-being and informed the time in nature categories (Supplementary material Fig. 1.8). Categories were 0, > 0–60 min, 60– < 120 min, 120– < 240 min, and > 240 min weekly time outdoors.

### GLM results: GBS associations with well-being

**Figure 1:**
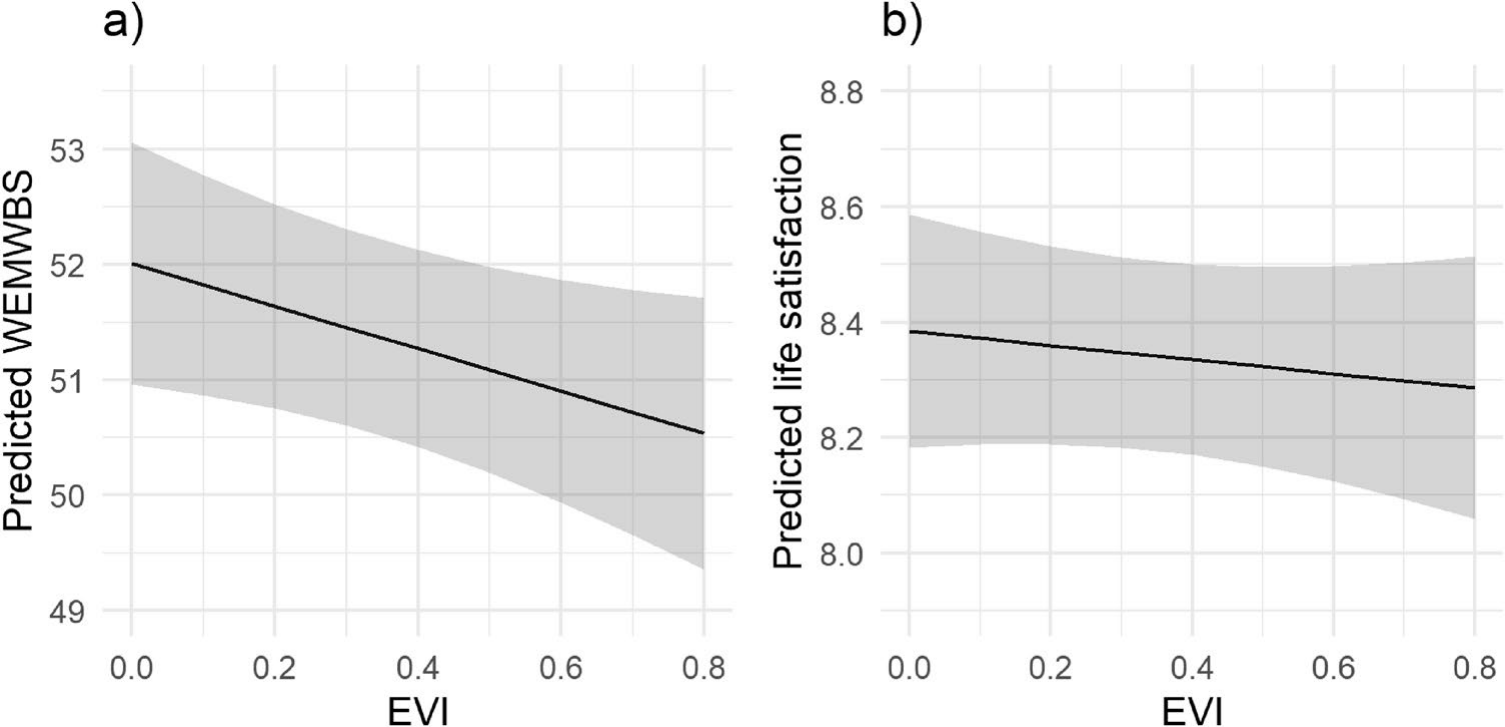
Predicted subjective well-being as measured by (**a**) WEMWBS and (**b**) life satisfaction. by neighbourhood green-ness (EVI). Predictions are based on fully adjusted models (see “Methods and data”) with fixed covariates. The y axes have been scaled to represent approximately equivalent proportions of the range (~ 8%).

In fully adjusted generalised linear models, there was an inverse association between residential EVI and WEMWBS (β =  − 1.84, 95% CI − 3.63, − 0.05; Fig. 1a), and this was also the case for urban residents only in a stratified model (β =  − 3.12, 95% CI − 5.76, − 0.49; Supplementary Table 2.2). EVI was not related to WEMWBS in models that were either unadjusted, or adjusted for all covariates except urban status. EVI was positively associated with life satisfaction in unadjusted models only (β = 0.32, 95% CI 0.03, 0.61) but there was no evidence of association with life satisfaction in either adjusted models (β =  − 0.12, 95% CI − 0.47, 0.22; Fig. 1b) or stratified for those in urban areas only (Supplementary Table 2.3).

**Figure 2:**
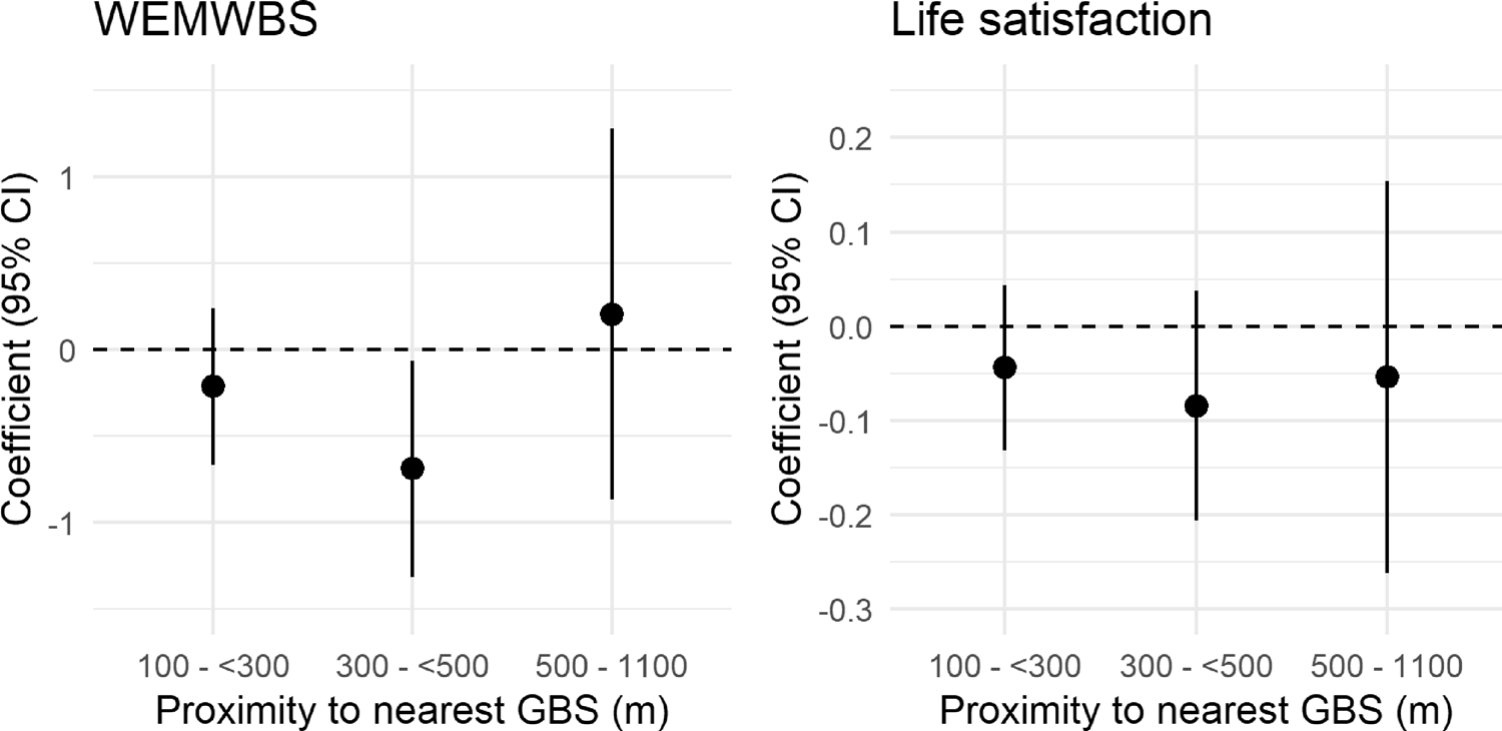
Coefficients for association between WEMWBS and life satisfaction with proximity to nearest GBS from the home versus reference category (< 100 m). Coefficients result from fully adjusted generalised linear models (see “Methods and data”). The y axes have been scaled to represent approximately equivalent proportions of the range (~ 5%) for each outcome scale. Full model results in Supplemental Tables 2.4 and 2.5.

Living 500–1100 m (*vs.* < 100 m) from the nearest GBS was associated with higher WEMWBS in the unadjusted model only (β = 1.46, 95% CI 0.36, 2.57). After adjustment, there were no clear trends in WEMWBS with increasing distance to GBS. In fully adjusted models, living 300–500 m from the nearest GBS was found to be associated with lower WEMWBS (*vs.* < 100 m, β =  − 0.69, 95% CI =  − 1.32, − 0.06; Fig. 2). For those living in urban areas only, living 100– < 300 m from the nearest GBS was associated with lower WEMWBS (*vs*. < 100 m, β =  − 0.60, 95% CI − 1.17, − 0.02), although categories at greater distances were not related (Supplementary Table 2.4). Proximity to nearest GBS was not found to be associated with life satisfaction in any of the models (Fig. 2 and Supplementary Table 2.5).

**Figure 3:**
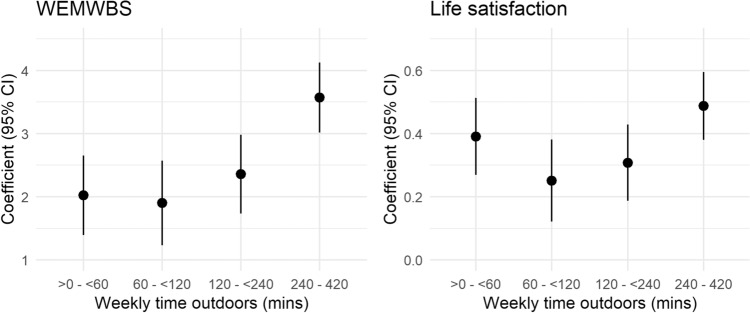
Model coefficients for weekly time in nature associated with subjective well-being for both WEMWBS and life satisfaction (reference category = no time outdoors). Coefficients are for fully adjusted generalised linear models predicting WEMWBS (left) and life satisfaction (right). The y axes have been scaled to represent approximately equivalent proportions of the range (~ 6%). Full model results in Supplemental Tables 2.6, 2.7.

Weekly time in nature was consistently positively associated with higher subjective well-being as measured by both WEMWBS (fully adjusted, 4–7 h *vs* 0: β = 3.57, 95% CI 3.02, 4.13) and life satisfaction (fully adjusted, 4–7 h *vs.* 0: β = 0.49, 95% CI 0.38, 0.59; Fig. 3). There were higher well-being scores as the weekly time in nature reached 60 min and more. However, spending at least a little time (> 0– < 60 min) in nature each week was associated with a higher than expected coefficient considering the subsequent trend. This was particularly the case for life satisfaction (Fig. 3; Supplementary Tables 2.6, 2.7).

#### Moderation by deprivation

When an interaction term was included between the residential exposure measures and deprivation status, there was no evidence that deprivation status moderated the association between either EVI or proximity to the nearest GBS and subjective well-being, for either well-being measure (e.g. EVI x In deprivation *vs.* EVI x not, WEMWBS: β = − 1.92, 95% CI − 6.35, 2.51; Supplementary Table 2.8). Likelihood ratio tests (LRTs) comparing models with and without interaction terms were non-significant for all combinations of residential exposure and subjective mental well-being (e.g. LRT; EVI and WEMWBS, χ^2^ = 0.72, *p* = 0.395; Supplementary Table 2.8), suggesting that adding interactions did not result in significant differences between the models.

**Figure 4:**
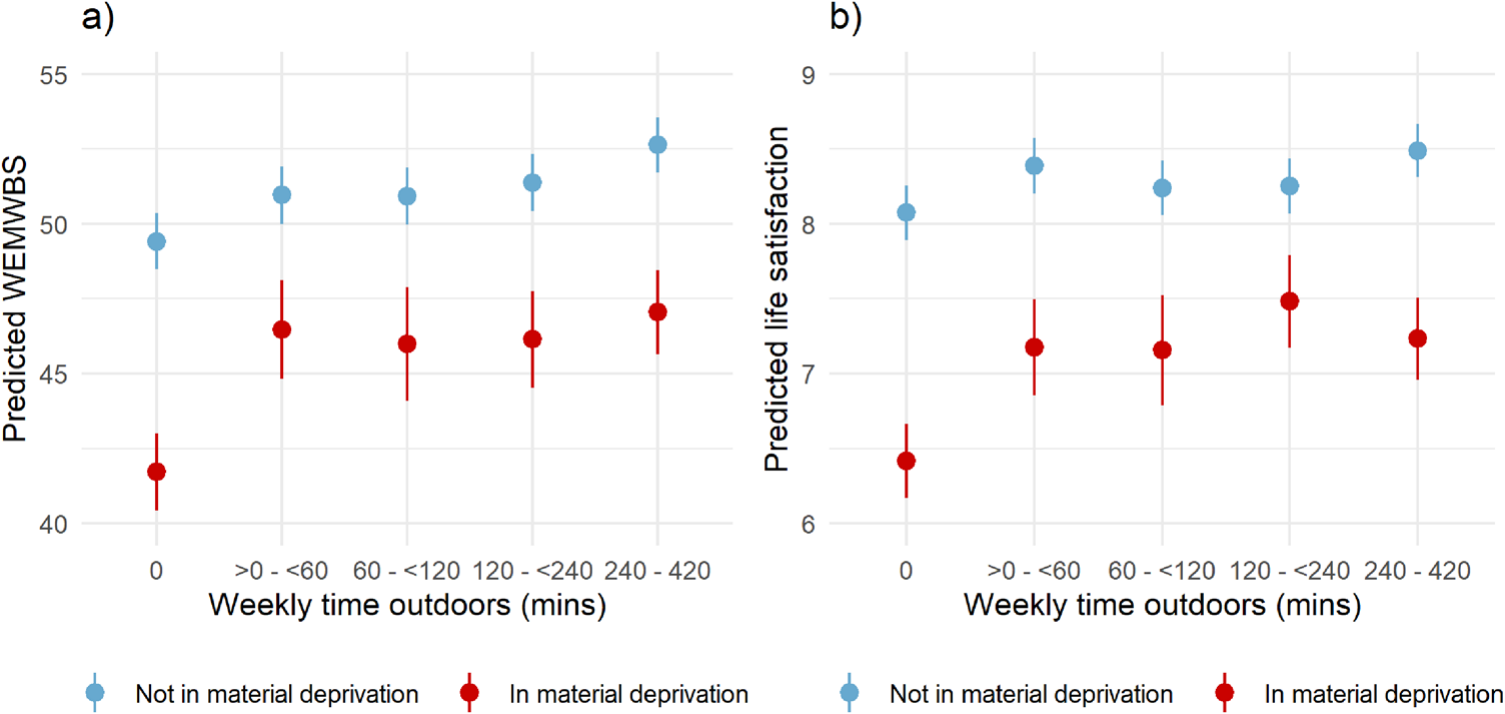
Predicted subjective well-being (WEMWBS and life satisfaction) by time in nature by deprivation status. Predictions are based on fully adjusted model results (see “Methods and data”) with fixed covariates. The y axes have been scaled to represent approximately equivalent proportions of the range (~ 27%). Full model results are in Supplementary Table 2.8.

There was evidence of interactions between weekly time in nature and deprivation for both WEMWBS and life satisfaction, indicating that deprivation status moderated the relationship between time in nature and subjective well-being (e.g. LRT; Time in nature and WEMWBS, χ^2^ = 17.82, *p* = 0.001; Supplementary Table 2.8). For those not in material deprivation, both WEMWBS and life satisfaction were generally higher, and there was a general gradual increase in subjective well-being with increasing time in nature. Those in material deprivation and spending no time in nature weekly reported considerably lower subjective well-being in comparison to those not in material deprivation. On average, with no time in nature, being in deprivation was associated with subjective mental well-being scores that were 7.7 lower for WEMWBS and 1.7 lower for life satisfaction, compared to those not in deprivation (Supplementary Table 2.8). However, when spending more than zero minutes, but less than one hour outdoors weekly (> 0 to < 60 min), this inequality was reduced to − 4.5 for WEMWBS and to − 1.22 for life satisfaction (Fig. 4 and Supplementary Table 2.8).

## Discussion

We explored the relationships between different measures of nature exposure and mental well-being. We did not find evidence to support hypothesis 1a. In contrast, we found that residential green-ness was negatively associated with mental well-being (as measured by WEMWBS). We did not find evidence that residential green-ness was related to life satisfaction. There was some indication that living further from the nearest GBS was related to lower levels of WEMWBS, although there were no clear trends across distance categories. Living 300–500 m from the nearest GBS was related to lower levels of WEMWBS (*vs.* < 100 m) for the full sample and 100–300 m from the nearest GBS was related to lower levels of WEMWBS (*vs.* < 100 m) for those in urban areas only. Again, there was no association with the one-item life satisfaction and proximity to nearest GBS. Supporting hypothesis 1b, spending more time in nature was consistently related to well-being, for both WEMWBS and life satisfaction measures.

Contrary to hypothesis 2a, we found no evidence that deprivation status moderates the relationship between residential GBS and well-being. However, supporting hypothesis 2b, we did find evidence that household material deprivation status moderates the relationship between time in nature and well-being in support of the equigenesis theory. This means that well-being inequalities by material deprivation were lower amongst people spending any time in nature compared to those spending no time.

Our finding that residential green-ness is negatively associated with well-being is in contrast with other results in the literature. In China, greenery as measured with both NDVI and streetscape greenery, were related to higher well-being (World Health Organisation 5-item index)^52^. And, in the Australian cities of Sydney and Melbourne, green-ness (NDVI) was associated with both the WHO-5 and a multi-item life satisfaction index (although not the two New Zealand cities in the study)^53^. Well-being as measured using WEMWBS was also found to be positively related to NDVI in a 250-m buffer for respondents to a survey in England^36^. However, two of these studies used non-random and non-representative samples^36,53^. Further studies link green-ness with mental health measures including in Canada^54^, and England^55^ with lower odds of poor mental health outcomes with increasing residential green-ness. However, other studies have also found no or mixed associations. Residential availability (surrounding green-ness and presence/absence of GBS) was not related to any mental health outcome in four European cities^20^ and in England, greenspace coverage (of a residential administrative unit) was not related to well-being (a short version of WEMWBS) once an urban/rural covariate was included^56^.

There is a lack of studies linking coastal proximity with well-being specifically, however, proximity to the coast has been found to be related to positive mental health in England^41,57^. Many of Wales’s largest urban areas (e.g. Swansea, Cardiff, Newport) are close to the coast (although there are exceptions), such that these urban areas with correspondingly low EVI are also often relatively close to the coast and associated well-being benefits. It is possible that the omission of blue spaces specifically contributes to the observed inverse relationship between EVI and well-being.

It is somewhat surprising that proximity to the nearest GBS was not found to be consistently related to well-being, particularly when proximity has been found to be related to visit frequency^58,59^. Further, the measure used here is considered to be relatively accurate as it follows the road/path network rather than a straight-line distance^60^. Our results may reflect the high proportion of people within our sample (> 80%) who lived within 300 m of their nearest GBS. Only 4% of our sample lived further than 500 m from their nearest GBS. However, the GBS included are of various sizes including small spaces, such as grass verges, and these may therefore not be reflective of the locations individuals visit. Further, people may visit GBS other than that closest to their home^61^. For example, for a sample of residents of Sheffield, UK, who self-selected to download an app, the mean trip distance to green spaces from home ranged from ~ 1200– ~ 1400 m, although these visits include incidental visits and did not include visits outside the city^62^. While, in Börger, et al.^63^, the average round trip distance for visits to blue spaces (most recent) was 24.80 km for nearly 6000 respondents to the BlueHealth International Survey in 14 European countries.

We found consistent positive relationships between time spent in nature and both measures of well-being. Our findings of a lack of a relationship for residential green-ness, but a positive relationship for time in nature and well-being, are consistent with a European four city study conducted by Triguero-Mas, et al.^20^ that used more objective measures of time in nature using GPS tracking. Other previous findings have been more mixed. For instance, a systematic review on this issue^34^ found only four ‘good’ quality studies included in the review (all cross-sectional), one found positive associations with time in nature and mental health and vitality domains of the SF-36 in four European cities^64^, one found a positive relationship in England for eudaimonic well-being (feeling life is worthwhile) but not life satisfaction using the same metric as in the current study^65^, and two studies conducted in Scotland found no association including using the WEMWBS as used here^38,39^.

Our findings are similar to those from a comparable study using a survey conducted in England, although with key differences. White et al.^24^ also analysed associations between self-reported time in nature and life satisfaction using a nationally (for England) representative recreation survey. This study suggested that individuals needed to spend at least 120 min per week in natural environments (both green and blue spaces, in urban and rural environments) to have higher life satisfaction relative to those who spent no time in nature. Whereas our findings in Wales suggested that those who spent any time in nature (> 0 min per week) also had significantly higher life satisfaction. These differences may be the result of different methodological and statistical approaches and/or different strengths in the relationships across the two countries. However, they are consistent in showing that spending time in nature is associated with higher well-being.

Our findings provide evidence in support of the equigenesis hypothesis, that the association between time spent visiting GBS was larger for those who were deprived relative those who were not, such that inequalities in well-being were narrowed. As Mitchell, et al.^42^ suggest, it is feasible that those under greater stress—such as those living in materially deprived circumstances—may benefit more from the stress recovery and restorative potential of time spent in natural environments. Contrary to Mitchell, et al.^42^, however, we did not find moderation between accessibility and well-being. Instead, we found moderation of actual use, which was not measured in Mitchell et al.^42^. Indeed, most other studies exploring similar questions have also focused on area indicators rather than recreational time^41,66–68^.

The variation observed in our results, and in the wider literature, may be at least partly due to the variation in the methods used to assess exposure to nature and mental health and well-being outcomes. Quantity can be measured in terms of area coverage, density or ‘green-ness’, typically within a radius around the home or administrative unit^69^, based on either satellite imagery or established landcover maps^49,54,55,67,70,71^. More recently, ‘street-view’ perspectives have been applied^72^. Access is typically measured in terms of proximity to GBS^41,73–75^, either as a Euclidian distance, or via road/path networks^60^, although public accessibility cannot be assumed. The nature of some blue spaces (e.g. linear features such as the coast) means they are perhaps more often measured in terms of proximity rather than area coverage^41,73–75^. Measures of the use of GBS also vary^27^, and can include recreational visit frequency to specific spaces or types of environment (e.g. Refs.^23,37,65,76^); time spent recreating in nature within a specific period of time^24,77–79^; as well as incidental use (e.g. commuting^23^). For residents of Wales, our findings suggest that actual use of GBS seems to be more strongly associated with well-being than measures of GBS access and quantity, although these findings are subject to design and sample limitations.

A key strength of this study was the nationally representative nature of the survey and the unique anonymised linkage of respondents to data on high-resolution residential GBS exposure measures within the SAIL databank. Linked data permitted the analysis of residential GBS exposure at much higher spatial resolution than is typically possible, with address-level geolocation coupled with road/footpath network proximity analysis. These GBS data are based on a large, integrated, national-scale spatial dataset of potentially accessible spaces enabling the identification of the closest accessible space for analysis.

A further strength was the exploratory analyses using GAMs, which permitted the nature of associations to be assessed before implementation of more traditional GLMs developed to reflect the underlying patterns in the data with interpretable model results. Exploring the robustness across two conceptually different well-being outcomes was also an advantage. Assessment of effect modification by socio-economic status was able to make use of a composite individual/household-level indicator, more precisely characterising individual level deprivation compared to the often used small-area deprivation indicators.

The most important limitation is the cross-sectional data used here. Although the wider project^29^ did have home exposure and health data on the same individuals over time, the NSW data on recreational visits was collected from individuals cross-sectionally. We therefore cannot confirm causation nor the direction of the associations. For example, these results may indicate that people with higher well-being spend more time outdoors. Whilst it does not discount this possibility, previous multi-country work has suggested that people taking medication for anxiety and/or depression may actually visit nature as much or even more than those without such conditions^80^, potentially to help them self-manage their symptoms, suggesting that our results are unlikely to solely be reflecting people with poorer mental health not making these kinds of visits.

EVI is a general measure of green-ness and does not capture the diversity of environmental conditions in people’s home neighbourhoods. Similarly, our GBS proximity measure does not distinguish between different types, sizes or qualities of spaces, and therefore we would expect a fairly substantial degree of heterogeneity in what this exposure measure reflects, and opportunities afforded for recreation. Further covariates may have been of relevance for visitation and well-being, but could not be included in analyses due to lack of data such as dog ownership^81^ and time spent in private gardens^22,82^. It is also possible that there is a mediating relationship between household material deprivation and well-being through the mechanism of spending time in nature and this could be explored in further work.

Social desirability bias is possible in the responses to the NSW questions, in particular because data collection was carried out in person^83^. For instance, people may have over-reported visit frequency if they felt this is a socially acceptable norm. Therefore, we should be cautious about interpreting the absolute values until such reports can be compared with other methods (e.g. through the use of experience sampling protocols^84^). A further issue arises if different groups are more or less affected by such biases—e.g. people not in material deprivation may feel more pressured to report more visits believing that such visits are more normative for their social group. Although not impossible, we feel this is unlikely. Specifically, the NSW is a parallel survey to the Monitor of Engagement in Natural Environments (MENE) in England which used a similar sampling protocol, in-home interviews, and very similar questions. Results from the MENE found no consistent bias in specific social groups reporting the quantity of visits to nature. Rather, distinct patterns in frequency were found for visit location, e.g. with wealthier groups reporting visiting woodlands more often, and poorer groups reporting visiting urban parks and beaches more often^85^. Thus, although social desirability bias may play some role, we consider it unlikely to be systematically affecting respondents’ overall visits frequency reports as a function of household deprivation.

Further research is needed to understand the characteristics of the natural environments that are being used for leisure by residents of Wales that seem to be providing benefits. This could then inform efforts to extend the availability, accessibility and use of these beneficial spaces to a greater proportion of the population through, for instance, land access strategies or land management and planning policies, removing barriers to greenspace use in deprived communities, or interventions to encourage use^86–89^. The latter could include simple practical interventions such as improving toilet facilities in natural settings, as well as addressing more complex issues such as fear of crime or lack of knowledge of how to access greenspaces^89^. Further, quality of public green spaces may be negatively correlated with area deprivation^90^ and improvements to the quality of outdoor spaces that are available and accessible may increase their use^63^.

Further research could also include exploring the mechanisms between both green-ness and time in nature and well-being. For example, surveying people regarding physical activity in nature in detail^14^ or recording GPS and/or accelerometry^91–93^_._ Given the lack of relationship observed between proximity to the nearest GBS and well-being, we suggest a more detailed approach by including not only the proximity to the nearest GBS but also the type of the nearest GBS. Alternatively, different modelling approaches could be applied such as distance-decay methods^94^.

## Conclusion

Although we find negative or mixed results for associations between residential GBS amount and proximity and well-being, we find consistent positive associations between time spent in nature and well-being. To the best of our knowledge, we provide the first evidence that the association between time spent outdoors and well-being is moderated by household-level deprivation. Spending time in nature may therefore contribute towards the mitigation and/or prevention of mental health problems for those exposed to material deprivation. Whilst further research is needed to fully understand this relationship, policies enabling use of, and access to, GBS for leisure by more socio-economically deprived groups may be beneficial for equitable population mental health and well-being. Further, our findings add to the evidence that relationships between nature exposure and well-being outcomes are complex and may be non-linear.

## Methods and data

### Data

The current research was part of a larger project looking at green and bluespace exposure across Wales, which linked medical records of 2.3 million adults to GBS measures^29^ at the household level (1.498 million residences). This study was approved by the Secure Anonymised Information Linkage (SAIL) Information Governance Review Panel (project 0562) in Wales. All data were anonymised prior to access and analysis and the research was performed in accordance with relevant guidelines/regulations. As part of this exercise, we linked individuals to available survey information from the National Survey for Wales (NSW). The current research uses this subset of people who had both area data and self-reported use data from the NSW.

GBS access/proximity and ambient green-ness measures were available for NSW participants via linkage of NSW data via the Secure Anonymised Information Linkage (SAIL) Databank^28–30^.

The NSW (http://gov.wales/national-survey-wales) is commissioned by the Welsh Government and carried out by the Office for National Statistics (ONS). A Natural Resources Wales module of the NSW includes questions on environmental issues and use of outdoor spaces. For those who consented, NSW responses from individuals were linked into the SAIL Databank (as above). Data were derived from responses to survey data from 2016/17 (adults n = 8932) and 2018/19 (n = 10,937)^45,46^. Data from 2017/18 were not used because of differences in the Natural Resources Wales survey module in that year, with relevant questions not asked.

Full details of survey methodologies can be found in the NSW technical reports^95,96^. Briefly, NSW interviews were conducted by trained interviewers in person at participants’ homes. Residential addresses in Wales were randomly selected, stratified by local authority, LA, n = 22), with survey effort approximately proportional to LA populations. Only one participant (aged 16 +) per residential address was interviewed. Surveys took place throughout the year, so include responses across seasons. Certain modules of the questionnaire were only asked of a random sub-sample of total participants, including the Natural Resources Wales module. Survey weights were provided that compensate for differences in sampling probability between different types of household, and for differences between sub-sample and population profiles; analysis conducted by the NSW indicated that non-response bias was negligible and weights therefore do not adjust for this.

### Outcome measures

For the WEMWBS, participants were presented with a series of 14 positively-worded statements, such as ‘I’ve been feeling good about myself’ and ‘I’ve been thinking clearly’. They were asked to indicate how often they have had these thoughts or feelings within the last two weeks, giving responses on a five-category scale: ‘none of the time’, ‘rarely’, ‘some of the time’, ‘often’, or ‘all of the time’. These responses are scored from one to five and summed, giving an overall score between 14 and 70 (higher scores indicate more positive mental well-being).

Life satisfaction is a measure of evaluative well-being based on responses to the question, “Overall, how satisfied are you with your life nowadays?”^97^. Responses are scored 0–10, with zero being ‘not at all satisfied’ and 10 being ‘completely satisfied’.

### Exposure measures

Two measures of residential GBS (residential green-ness and access/proximity), and one measure of use (leisure time in nature) were considered as exposures in this analysis. Detailed methodology regarding the green-ness and access/proximity measures is provided in supplementary materials Sect. 3.

#### Residential Green-ness

The residential green-ness measure is estimated using satellite imagery as the annual mean Enhanced Vegetation Index (EVI) averaged using a 300 m buffer centred on each household location, clipped to the coastline. Although NDVI has been more commonly used in studies investigating the links between green-ness and well-being^3^, EVI was chosen as it reduces both atmospheric and soil background noise^98^. Values theoretically range from -1 to + 1 where negative values indicate a lack of vegetation. Typical values in broadleaf woodlands range from ~ 0.2–0.3 in winter to ~ 0.6–0.7 in summer^47^, and the mean EVI of a 500 m buffer around the home of a study in London (UK) was 0.37^99^. See supplementary materials Sect. 3 for full details.

#### Proximity to nearest GBS

Vector data from multiple sources were used to calculate access to GBS. These sources were: MasterMap (Topography Layer^100^), Local green spaces^101^; Local Government Audits (provided by 14 of 22 local authorities); the Lle geoportal^102^, and OpenStreetMap (OSM)^103^. See supplementary materials Sect. 3 and future papers for further details^104^. Potential GBS were categorised according to a pre-defined hierarchical typology to only include accessible blue or green spaces, this excluded farmland and gardens^104^. These confirmed GBS were then assigned access points snapped to the road and footpath network. Proximities to residences were calculated to a maximum distance of 1600 m and proximity to the nearest GBS was the final measure for GBS access. The maximum distance from any residence in the final sample to the nearest GBS was 1050 m.

#### Time in nature

An estimate of weekly leisure time spent in natural environments (‘time in nature’) was based on a number of questions about visits to GBS in Wales. The NSW survey specifies these visits as follows: “*The next questions are about outdoor recreation in Wales. We are interested in leisure visits and excursions to the Welsh outdoors of any length. These visits may have been made from your home or during holidays. By outdoors, we mean open spaces anywhere in the countryside or in towns and cities, including your local neighbourhood, paths, woodland, parks and farmland. Visits may have involved both active and passive pursuits.*” Questions used to derive time outdoors included those establishing which activities had been undertaken during these visits, and how long was spent doing them^45,46^. Due to heavy skewing in the distribution, this value was capped at 420 min per week (equivalent to an hour a day, see also Ref.^24^. Fifteen percent (of the final sample; n = 1125) of values were recoded this way.

### Covariates

Variables to be included in statistical models were selected on the basis of data availability and theoretical understanding of their potential importance and associations with either/both exposure and outcome variables (Table 2)^13,54,75,85,105,106^. The material deprivation measure is a derived binary indicator included in the NSW dataset; and the same as that used in the UK Family Resources Survey^43^. It is based on an additive score summarising whether the participant could afford a series of items, such as ‘a holiday away from home for at least a week a year’. These items differed for adults of pensionable age e.g. a warm home and access to a car or taxi when needed^46^. The series were scored and a threshold applied to assign binary categories of either in material deprivation or not^43^.

**Table 2:** Covariates included in modelling.

Variable (role)	Data source	Description	Continuous/categorical
Gender (confounder)	NSW	Male/Female (other responses too few for inclusion)	Categorical (two levels)
Age (confounder)	NSW	Individual age in years at time of survey; 16–24, 25–44, 45–64, 65–79, 80 +	Categorised
Economic status (confounder)	NSW	Employed; economically inactive (incl. full-time students and pensioners); unemployed	Categorical (three levels)
Material deprivation (confounder/modifier)	NSW	In material deprivation/not in material deprivation (see “Methods and data”)	Categorical (two levels)
Use of car (confounder)	NSW	Yes/No	Categorical (two levels)
Season (confounder)	NSW	Spring (Mar/Apr/May); Summer (Jun/Jul/Aug); Autumn (Sep/Oct/Nov); Winter (Dec/Jan/Feb)	Categorical (four levels)
Welsh index of multiple deprivation (WIMD) 2014 (confounder)	SAIL	Quintiles of rankings of LSOA^a^-level deprivation based on eight domains	Categorical (five levels)
Rural–urban category (confounder/modifier)	NSW	LSOA^a^-level classification derived from ONS^b^ data. Three categories: urban (> 10,000 people); town and fringe; village, hamlet and isolated dwellings	Categorical (three levels)
Local authority (confounder)	NSW	For proximity to nearest GBS only, local authority is also included to account for potential differences in GBS data provided by each local authority (not required for EVI or time in nature models)	Categorical

^a^Lower layer super output area (LSOA), the smallest area for UK statistics, designed to have similar population sizes of ~ 1500.

^b^Office for National Statistics (ONS).

### Data linkage

Data are linked via an individual-based unique “Anonymised Linking Field” (ALF) and household-level “Residential Anonymised Linking Fields” (RALFs)^107,108^. The anonymised linkage process permitted high spatial resolution (address-level) linkage of survey participants to GBS metrics for the area immediately around their home. Missing data were excluded (Table 3).

**Table 3:** Data joining and resulting sample sizes. Data were excluded where there were missing data.

Sample	Sample size	Excluded n
NSW full sample (2016/17 and 2018/19)	19,869	
Missing linkages	18,416	1453
NRW subsample	11,378	7038
Identifier dates match	10,744	634
With WIMD^a^ (joined by LSOA^b^)	10,706	38
With EVI	10,149	557
With time in nature	10,072	77
With outcome variables	8452	1620
With covariates: gender, economic status, age, and deprivation	8360	92
With GBS proximity	7631	729

^a^Welsh Index of Multiple Deprivation (WIMD).

^b^Lower layer super output area (LSOA).

### Analysis

Instead of making assumptions about the shape of the relationship, we took the approach of first applying generalised additive models (GAMs), to inform analysis using a generalised linear modelling (GLM) approach. GAMs are an extension of GLMs, which are highly flexible and do not assume linearity in the relationships between explanatory variables and the mean of the response^109^. The fitted relationships between explanatory variables and outcomes were plotted and used to inform the specification of GLMs (Supplemental materials Sect. 1). Results from GLMs are more straightforward to interpret and hence the GLM results are the focus here. To test for effect modification, interaction terms were included. All analyses were undertaken in RStudio (version 2021.09.0 + 351) with R (version 4.1.1^110^, using the ‘mgcv’ package^109^ for GAMS.

Models were weighted using NSW sampling design weights^95,96^, which were converted to frequency weights using the ‘rescale_weights()’ function in the ‘parameters’ R package^111^. The covariates described above were included in fully adjusted models. However, given the potential for over-adjustment, models were also run without inclusion of urban/rural categories. For example, urban/rural category correlates strongly with ambient green-ness by definition because urban areas are classified as such due to the density of residential population and built environment*.*

In order to visualise key findings (Figs. 1 and 4), regression coefficients from fully adjusted models were used to derive predicted well-being scores across GBS exposure scales based on a typical set of covariate values, these were: WIMD = Q3, gender = female, age = 16–24, economic status = employed, deprivation status = not in material deprivation, use of a car = yes, urban status = urban, season = Autumn and survey wave = 2016–17.

## Supplementary Information

Supplementary Information.

## Data Availability

The data used in this study were accessed through the SAIL Databank at Swansea University, Swansea, UK. All proposals to use SAIL data are subject to review by an independent Information Governance Review Panel (IGRP). Information on the application process can be found at: https://www.saildatabank.com/application-process.
