# Supplementary material for: Visiting nature is associated with lower socioeconomic inequalities in well-being in Wales
Source: Sci Rep. 2023 Jun 15;13:9684. doi: 10.1038/s41598-023-35427-7 (PMC10272170; doi:10.1038/s41598-023-35427-7)
Supplement: Supplementary file 1 — Supplementary Information. [file 41598_2023_35427_MOESM1_ESM.docx]

## Supplemental materials

1. **Generalised Additive Modelling**

Generalised additive models (with thin plate regression splines; “ts” smooth function) were the first step in the analysis process. These were used to explore the shape of the relationship between the key predictors and the outcomes. Analyses were carried out in RStudio (version 1.4.1103) with R (version 4.1.0) and packages “mgcv” (Wood 2021, version 1.8-36) and “gratia” (version 0.6.0). An effective degrees of freedom (edf) of 1 indicates a linear relationship, while an edf of 2 indicates a quadratic relationship.

Table 1.1 summarises the approach used to model predictors in the main analyses based on the GAMs. This is followed by details of the results from these analyses.

Table 1.1 Summary of the approach used to model the predictors by exposure type and outcome in further GLM analyses

| Predictor | Outcome | Modelling terms |
| --- | --- | --- |
| EVI | WEMWBS | Linear |
| EVI | Life satisfaction | Linear |
| Proximity to nearest GBS | WEMWBS | Categories; 0 – <100, 100 - <300, 300 - <500 and 500 m – 1,100 m |
| Proximity to nearest GBS | Life satisfaction | Categories; 0 – <100, 100 - <300, 300 - <500 and 500 m – 1,100 m |
| Time outdoors | WEMWBS | Categories 0, >0 - <60, 60 - <120, 120 - <240, 240 -420 |
| Time outdoors | Life satisfaction | Categories 0, >0 - <60, 60 - <120, 120 - <240, 240 -420 |

- 1. *EVI predicting WEMWBS*

EVI was not significantly related to WEMWBS (edf = 0.258, *p* = 0.247) (Fig. 1.1). The GAM indicated no significant relationship between EVI and WEMWBS for those in material deprivation (edf = 0.58, *p*= 0.126) and for those not in material deprivation (edf = 0.62, *p* = 0.108; Fig. 1.2).

For those in urban areas, the GAM indicated a significant approximately linear negative relationship between EVI and WEMWBS (edf = 0.87, *p* = 0.009), while for those in village, hamlet and isolated dwellings and town and fringes there was no relationship (T&F, edf = 0.01, *p* = 0.932; V/H/I, edf = 0.01, *p* = 0.699, Fig. 1.3).

- - 1. GLM modelling decision

EVI predicting WEMWBS was modelled as linear term in subsequent generalised linear models (GLMs).


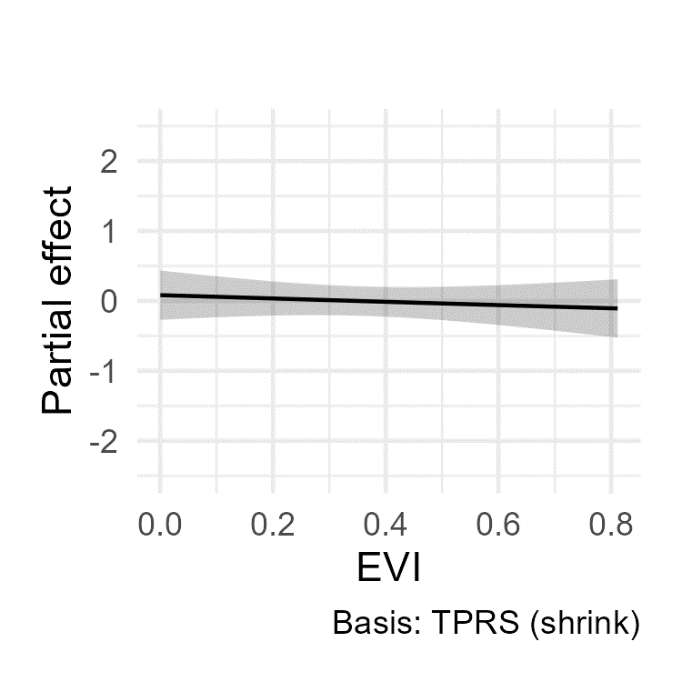


Figure 1.1 The smoothed function of EVI on WEMWBS. Model includes WIMD, gender, age, economic status, material deprivation, car use, season and wave.


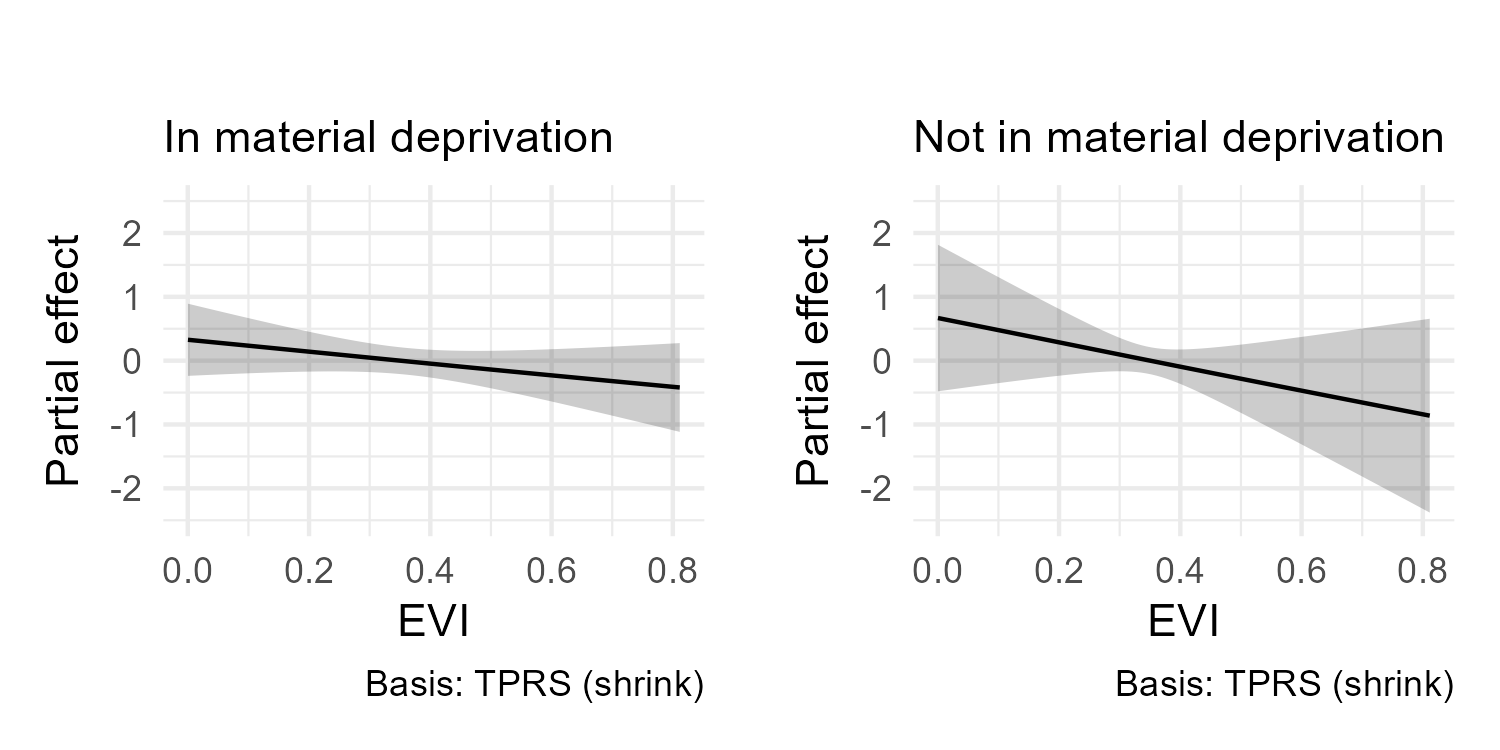


Figure 1.2 The smoothed function of EVI on WEMWBS by deprivation. Model includes WIMD, urban status, gender, age, economic status, material deprivation, car use, season and wave.


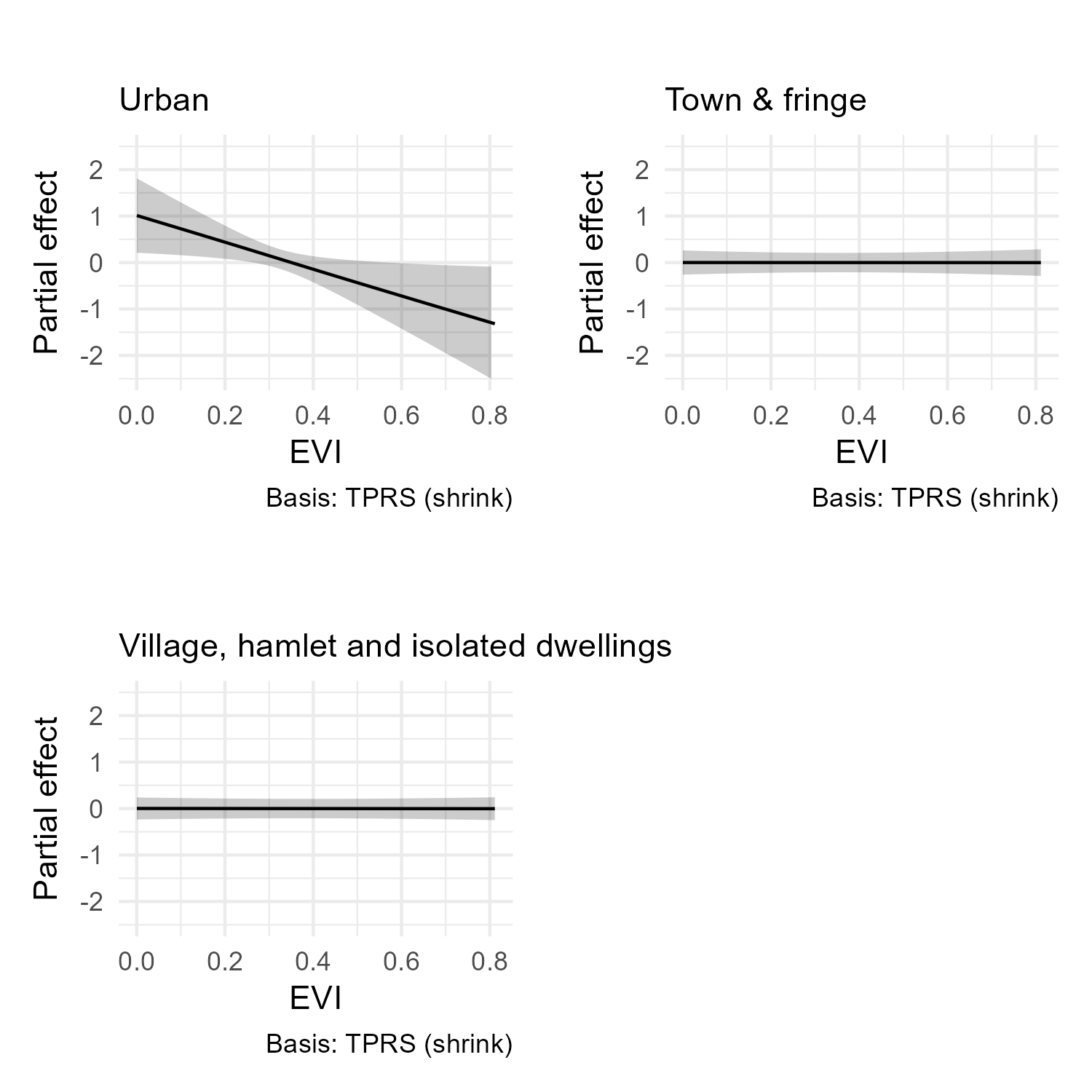


Figure 1.3 The smoothed function of EVI on WEMWBS by urban status. Models include WIMD, gender, age, economic status, material deprivation, car use, season and wave.

- 1. *EVI predicting life satisfaction*

EVI was also not found to be significantly related to life satisfaction (edf = 0.01, *p* = 0.646). However, it was significantly related for those in material deprivation (complex and “wiggly”, edf = 7.14, *p*<0.001; Fig. 1.4) but not significant for those not in material deprivation with a negative relationship (edf = 0.31, *p*=0.231). There were also no significant relationships by urban status (Urban: edf =0.02, *p* = 0.369; Town & fringe: edf =0.04, *p* = 0.364; Village, hamlet & isolated dwellings: edf = 2.43, *p* = 0.144; Fig. 1.5).

- - 1. GLM modelling decision

To maintain consistency with the WEMWBS modelling, EVI was also modelled as linear when predicting life satisfaction


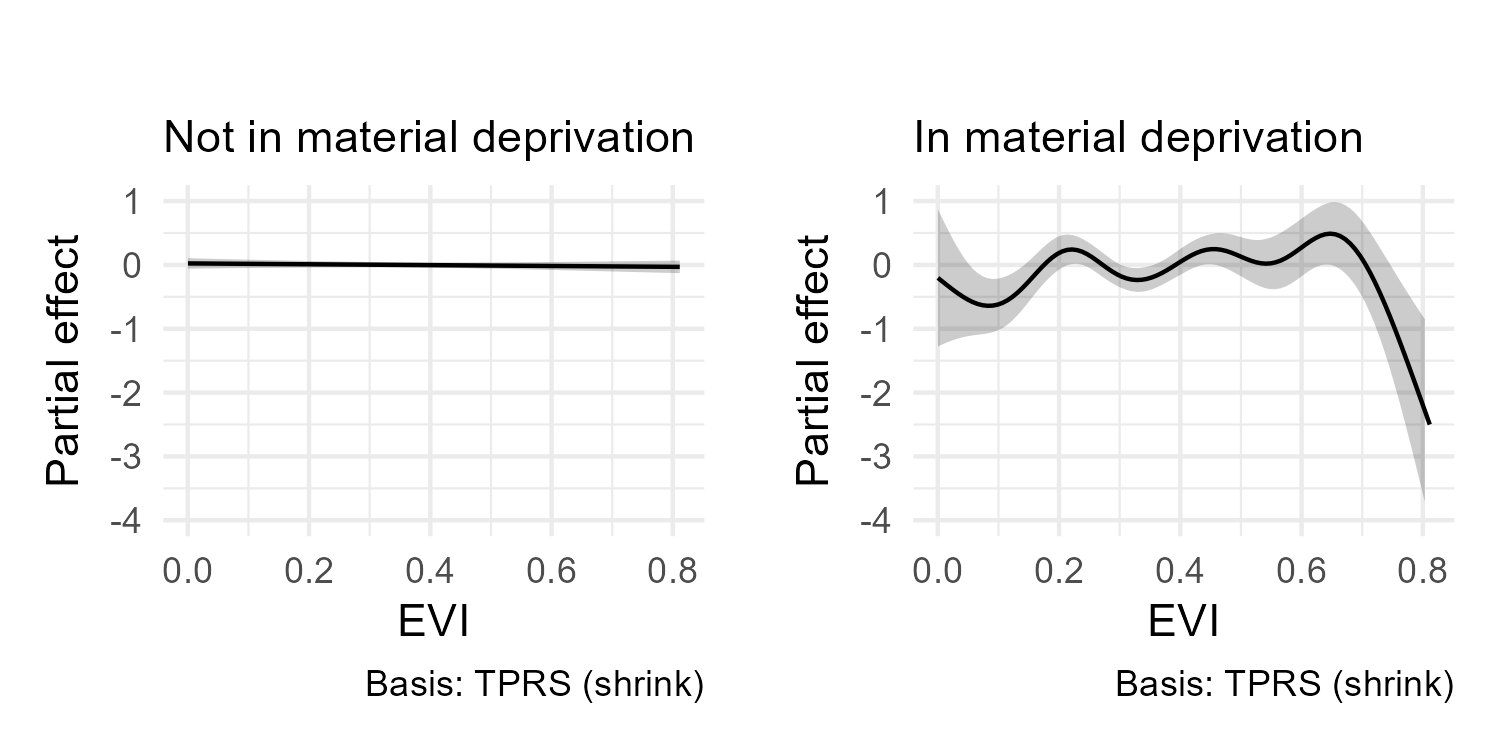


Figure 1.4 The smoothed function of EVI and the effect on life satisfaction by deprivation status. Model includes WIMD, gender, age, economic status, material deprivation, car use, season and wave.


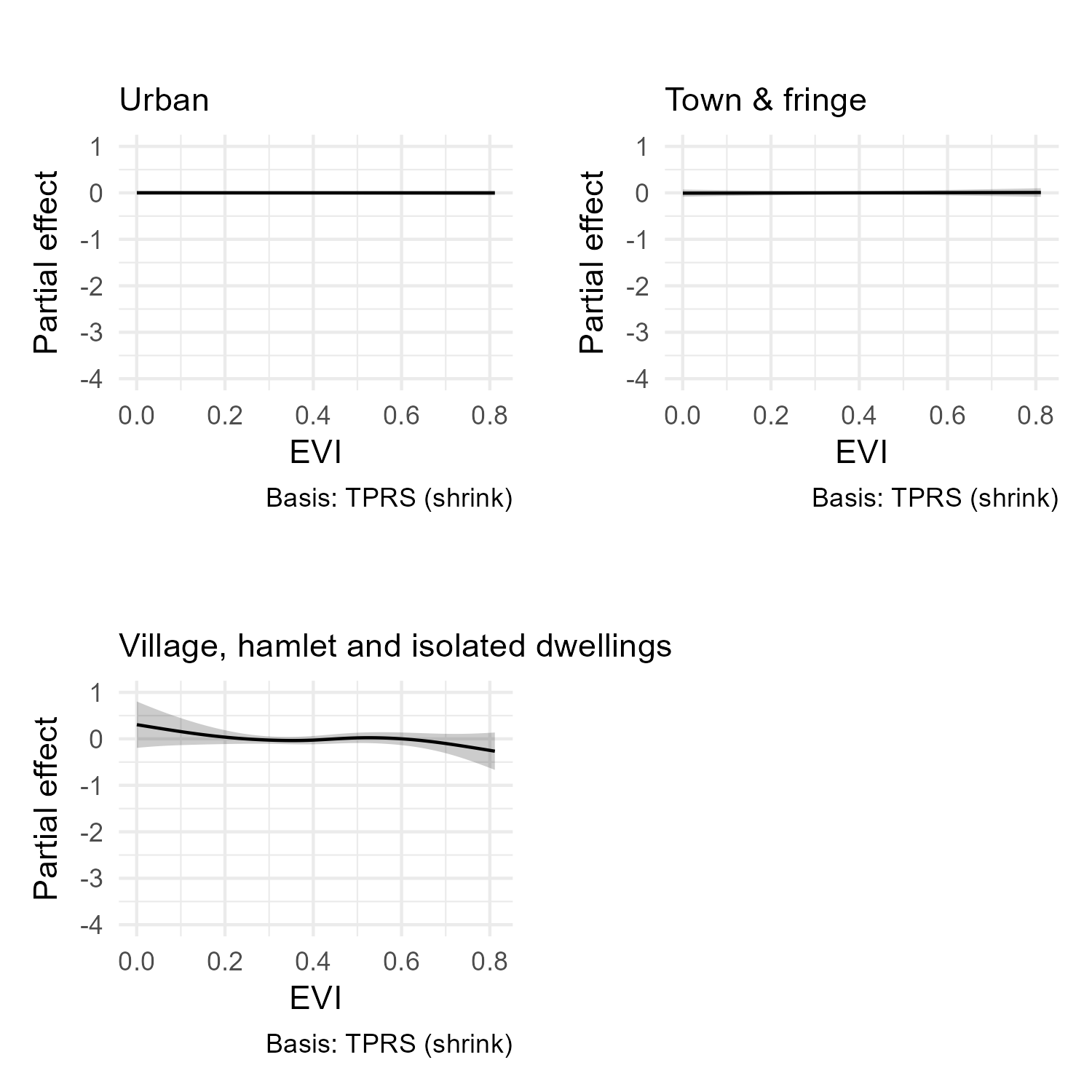


Figure 1.5 The smoothed function of EVI and the effect on life satisfaction by urban status (only Urban displayed). Model includes WIMD, gender, age, economic status, material deprivation, car use, season and wave.

- 1. *Proximity to nearest green or blue space predicting WEMWBS*

Proximity to nearest green or blue space was not found to be related to WEMWBS (edf = 0.01, *p* = 0.926). By urban status, proximity was not related to WEMWBS for those in either urban areas or town and fringe (Urban: edf = 0.01, *p* = 0.658; Town & fringe: edf <0.01, *p* = 0.673). For those living in village, hamlets and isolated dwellings the GAM indicated a complex relationship between proximity and WEMWBS (edf = 4.90, *p* < 0.001, Fig. 1.6).

By material deprivation, for those not in material deprivation proximity to nearest GBS was not related (edf = 0.02, *p* = 0.873) and for those in material deprivation the relationship was complex and significant (edf = 5.40, *p* <0.001).

- - 1. GLM modelling decision

Categories were used in further GLM analyses which we categorised based on visually inspecting the relationships for both identified complex relationships (Fig. 1.6) and adjusting cut-off points to the nearest 50 m. These were 0 – <100, 100 - <300, 300 - <500 and 500 – 1,100 m.


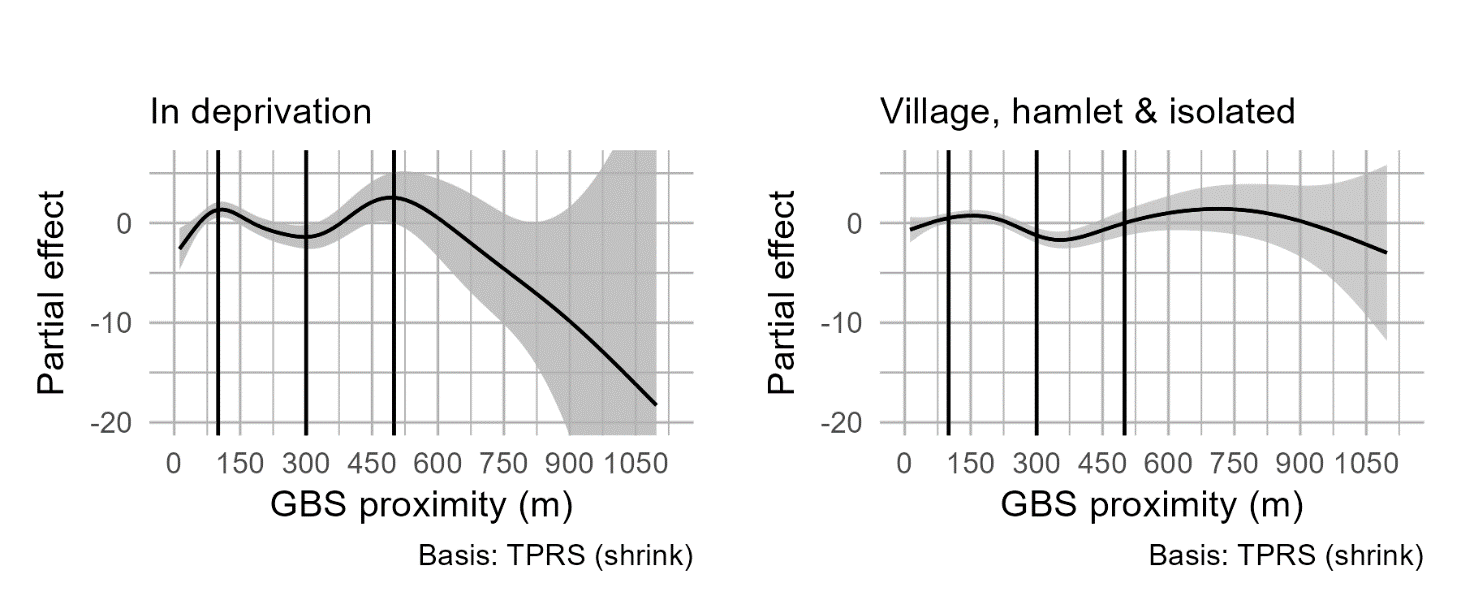


Figure 1.6 The smoothed function of proximity to nearest GBS and the effect on WEMWBS by a) urban status (only village, hamlet and isolated dwellings displayed) and b) deprivation status (only in deprivation displayed). Model includes WIMD, gender, age, economic status, material deprivation, car use, season, wave and LA. Vertical lines indicate the categories used in further analyses.

- 1. *Proximity to nearest green or blue space predicting life satisfaction*

Proximity to nearest green or blue space from the home was not found to be related to life satisfaction (edf = 0.02, *p* = 0.529). With separate relationships calculated by urban status, there was a marginally significant, approximately quadratic, relationship between proximity and life satisfaction for those in living in town and fringe (edf = 1.98, *p* = 0.058) and no significant relationships for those in village, hamlet and isolated dwellings (edf = 0.49, *p* = 0.164) or in urban areas (edf = 0.02, *p* = 0.635). For those not in deprivation, proximity to GBS was not related to life satisfaction (edf = 0.02, *p* = 0.624) while for those in deprivation, the relationship was significant and complex (edf = 5.01, *p*<0.001, Fig. 1.7).

- - 1. GLM modelling decision

Given the complex nature of the relationship between GBS proximity and life satisfaction for those in deprivation and a preference for maintaining consistency between models, the same categories as used in modelling GBS proximity and WEMWBS were used in further GLM analyses for life satisfaction.


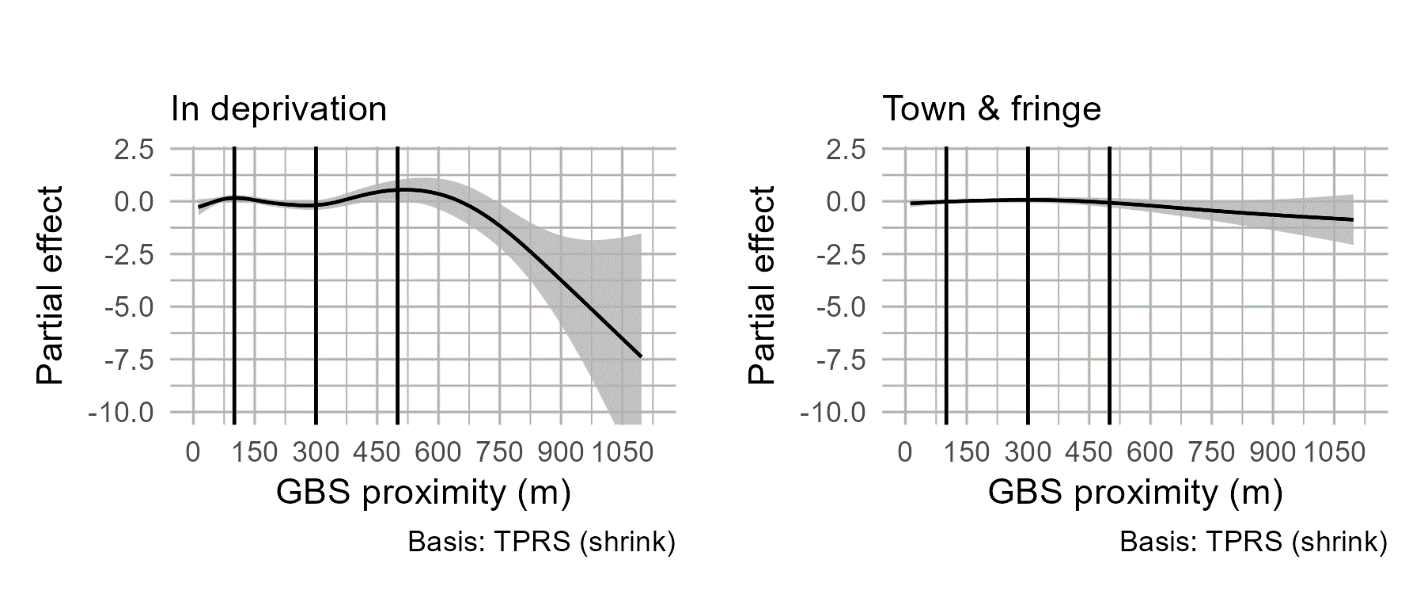


Figure 1.7 The smoothed function of proximity to nearest GBS and the effect on life satisfaction by a) urban status (only town & fringe displayed) and b) deprivation status (only in deprivation displayed). Model includes WIMD, gender, age, economic status, material deprivation, car use, season, wave and LA. Vertical lines indicate the categories used in further analyses.

- 1. *Time outdoors predicting WEMWBS*

Time outdoors was significantly related to WEMWBS with a complex relationship but generally positive (edf = 5.93, *p*<0.001; Fig. 1.8). For those not in material deprivation, the relationship was approximately linear (edf = 1.24, *p*<0.001) and for those in material deprivation, the relationship was complex and significant (edf = 3.43, *p<*0.001, Fig. 1.9).

Similarly, time outdoors was significantly associated with life satisfaction with a complex relationship (edf = 6.21, *p* <0.001, Fig. 1.8). For those not in material deprivation, the relationship was approximately linear (edf = 1.10, *p* <0.001) and for those in material deprivation, the relationship was more complex (edf = 2.58, *p*<0.001, Fig. 1.10).

- - 1. Modelling decisions.

Time outdoors was modelled categorically with categories of 0, >0 – 60 mins, 60 - <120 mins, 120 - <240 mins, and >240 mins weekly time outdoors.


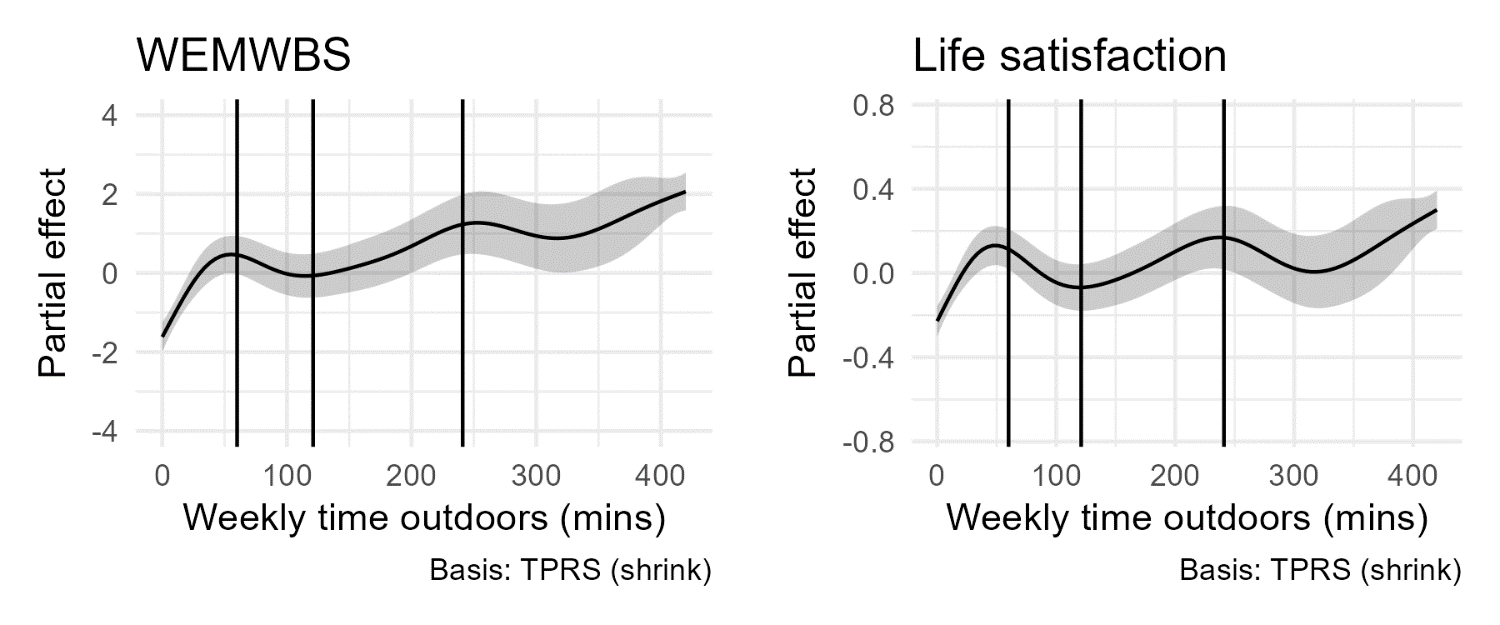


Figure 1.8 Weekly time outdoors predicting a) WEMWBS and b) life satisfaction


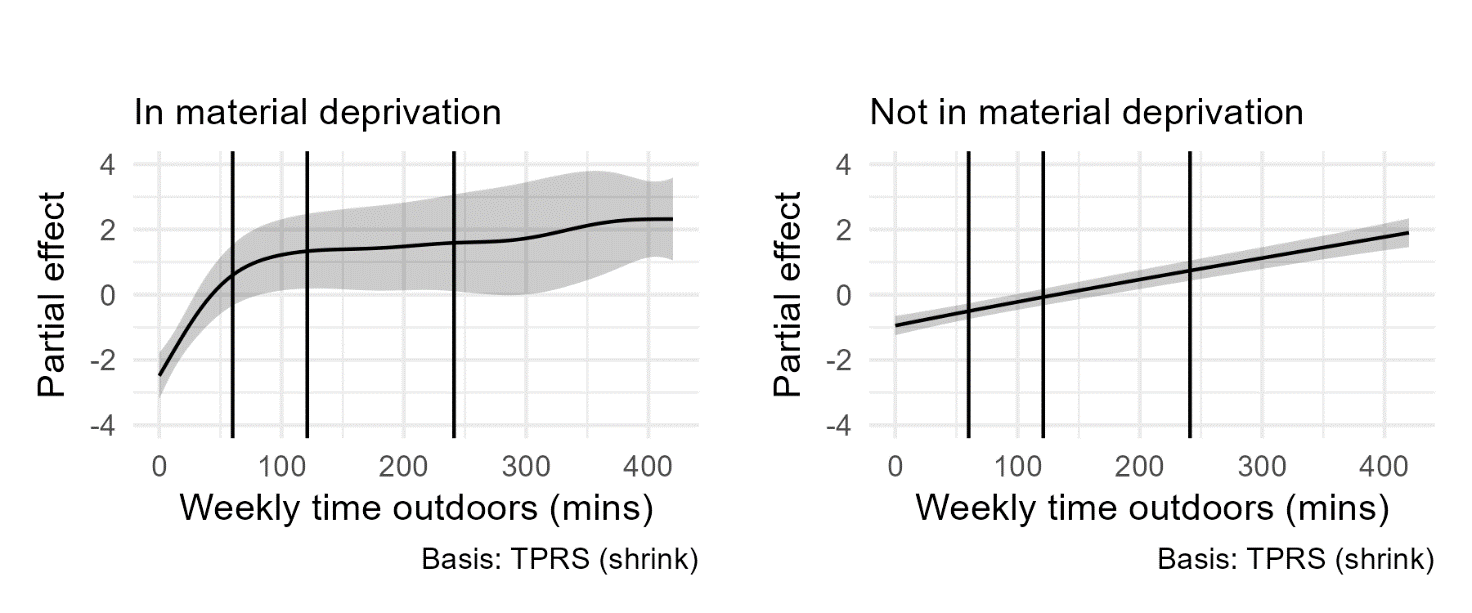


Figure 1.9 Weekly time outdoors predicting WEMWBS for those in material deprivation and those not in material deprivation.


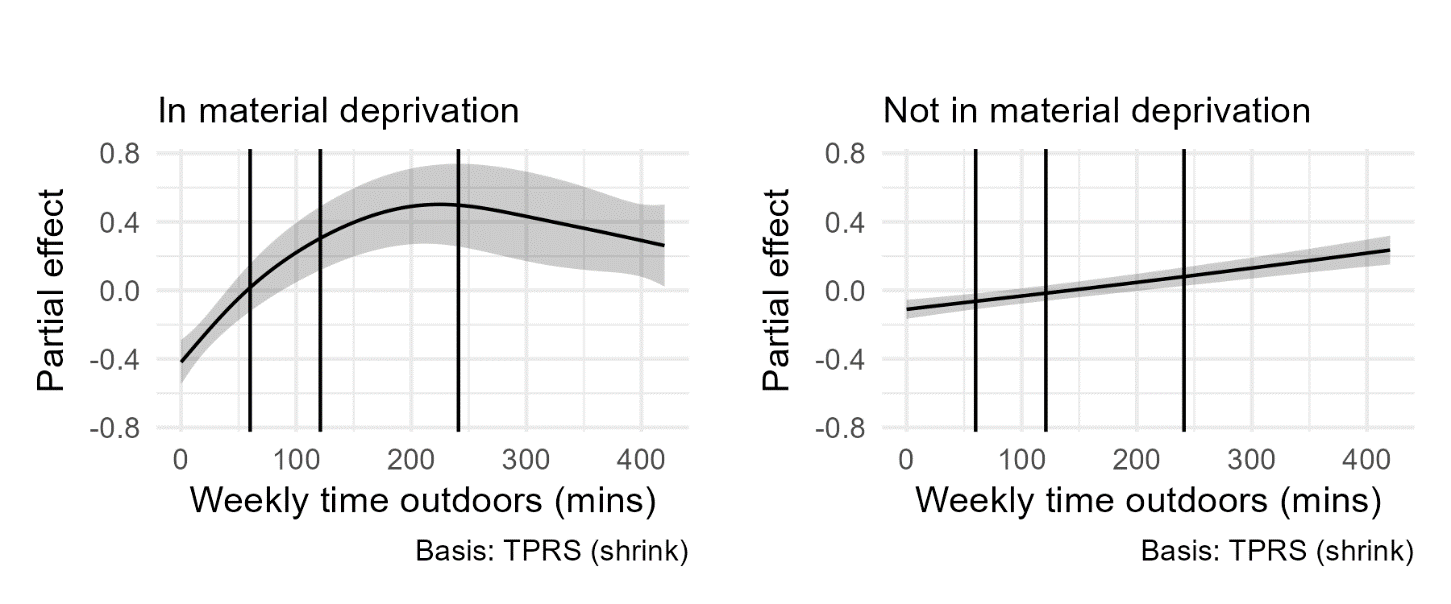


Figure 1.10 Predicting life satisfaction by weekly time outdoors for those in material deprivation and those not in material deprivation

1. **Results tables**

Table 2.1 Sample descriptives

|  |  |  | **WEMWBS** | | **Life satisfaction** | |
| --- | --- | --- | --- | --- | --- | --- |
| **Category** | **Counts** | **Weighted %** | **Mean** | **SD** | **Mean** | **SD** |
| Full sample | 7631 |  | 50.92 | 9.36 | 7.77 | 1.84 |
| EVI |  |  |  |  |  |  |
| *0 - <0.2* | 815 | 10.02 | 50.92 | 9.17 | 7.71 | 1.79 |
| *0.2 - <0.4* | 4330 | 56.26 | 51.15 | 9.14 | 7.82 | 1.81 |
| *0.4 - <0.6* | 2100 | 28.22 | 51.19 | 9.28 | 7.93 | 1.69 |
| *0.6 - <0.82* | 386 | 5.50 | 52.46 | 8.05 | 7.92 | 1.61 |
| GBS proximity (m) |  |  |  |  |  |  |
| 0 - <100 | 2189 | 29.53 | 51.15 | 9.63 | 7.85 | 1.79 |
| 100 - <300 | 3957 | 51.28 | 51.19 | 9.03 | 7.83 | 1.76 |
| 300 - <500 | 1197 | 15.31 | 51.02 | 8.78 | 7.84 | 1.76 |
| 500 - 1100 | 288 | 3.88 | 52.61 | 7.79 | 8.00 | 1.69 |
| Weekly leisure time outdoors (mins) |  |  |  |  |  |  |
| *0* | 2309 | 26.19 | 48.89 | 10.25 | 7.46 | 2.14 |
| *>0 - <60* | 1210 | 16.25 | 51.30 | 8.54 | 7.98 | 1.62 |
| *60 - <120* | 953 | 13.51 | 51.30 | 8.16 | 7.89 | 1.62 |
| *120 - <240* | 1205 | 17.08 | 51.68 | 8.29 | 7.89 | 1.59 |
| *240 - 420* | 1954 | 26.97 | 53.06 | 8.82 | 8.08 | 1.55 |
| Urban status |  |  |  |  |  |  |
| *Urban* | 4702 | 60.27 | 50.88 | 9.24 | 7.79 | 1.79 |
| *Town & fringe* | 1129 | 15.29 | 51.46 | 9.15 | 7.82 | 1.78 |
| *Village, hamlet & isolated dwellings* | 1800 | 24.44 | 51.86 | 8.81 | 8.00 | 1.68 |
| WIMD |  |  |  |  |  |  |
| *Q1 Most deprived 20%* | 1201 | 14.31 | 49.11 | 10.51 | 7.51 | 2.07 |
| *Q2* | 1411 | 19.65 | 50.89 | 8.99 | 7.78 | 1.80 |
| *Q3* | 1730 | 23.71 | 51.09 | 8.99 | 7.89 | 1.71 |
| *Q4* | 1767 | 23.07 | 51.89 | 8.86 | 7.88 | 1.72 |
| *Q5 Least deprived 20%* | 1522 | 19.26 | 52.42 | 8.34 | 8.07 | 1.56 |
| Gender |  |  |  |  |  |  |
| *Female* | 4316 | 52.36 | 50.66 | 50.66 | 7.84 | 7.84 |
| *Male* | 3315 | 47.64 | 51.81 | 51.81 | 7.86 | 7.86 |
| Age |  |  |  |  |  |  |
| *16-24* | 435 | 12.75 | 50.60 | 8.49 | 7.92 | 1.59 |
| *25-44* | 1945 | 29.27 | 50.54 | 9.02 | 7.81 | 1.59 |
| *45-64* | 2651 | 34.17 | 50.95 | 9.57 | 7.68 | 1.94 |
| *65-79* | 2065 | 19.41 | 53.08 | 8.49 | 8.11 | 1.75 |
| *80+* | 535 | 4.39 | 51.15 | 9.84 | 7.96 | 1.83 |
| Economic status |  |  |  |  |  |  |
| *Employed* | 3777 | 56.18 | 51.62 | 8.28 | 7.93 | 1.52 |
| *Inactive* | 3686 | 41.59 | 50.89 | 10.05 | 7.79 | 2.01 |
| *Unemployed* | 168 | 2.24 | 46.77 | 10.15 | 6.76 | 2.12 |
| Deprivation status |  |  |  |  |  |  |
| *In material deprivation* | 1058 | 12.83 | 44.63 | 10.73 | 6.52 | 2.22 |
| *Not in material deprivation* | 6573 | 87.17 | 52.18 | 8.45 | 8.04 | 1.60 |
| Car access |  |  |  |  |  |  |
| *No* | 1048 | 10.16 | 47.39 | 10.70 | 7.14 | 2.21 |
| *Yes* | 6583 | 89.84 | 51.64 | 8.83 | 7.92 | 1.69 |
| Season |  |  |  |  |  |  |
| *Autumn* | 2093 | 24.76 | 51.34 | 9.12 | 7.95 | 1.72 |
| *Spring* | 1873 | 30.19 | 51.33 | 9.01 | 7.82 | 1.78 |
| *Summer* | 1537 | 23.89 | 51.46 | 9.19 | 7.79 | 1.77 |
| *Winter* | 2128 | 21.16 | 50.59 | 9.23 | 7.83 | 1.78 |
| Survey wave |  |  |  |  |  |  |
| *2016-17* | 4231 | 55.69 | 50.87 | 9.17 | 7.80 | 1.79 |
| *2018-19* | 3400 | 44.31 | 51.64 | 9.07 | 7.90 | 1.74 |

Table 2.2 Generalised linear model results predicting WEMWBS by EVI. Models include unadjusted with no covariates, adjusted with covariates, additionally adjusted with urban status, and adjusted for those residing in urban areas only.

|  | WEMWBS | | | | | | | |
| --- | --- | --- | --- | --- | --- | --- | --- | --- |
|  | Unadjusted | | Adjusted | | Adj. with urban | | Adj., urban only | |
| Characteristic | Beta^1^ | 95% CI^2^ | Beta^1^ | 95% CI^2^ | Beta^1^ | 95% CI^2^ | Beta^1^ | 95% CI^2^ |
| Intercept | **50.86***** | **50.29, 51.44** | **49.35***** | **48.16, 50.55** | **49.58***** | **48.36, 50.79** | **51.10***** | **49.57, 52.64** |
| EVI | 0.97 | -0.54, 2.48 | --0.92 | -2.49, 0.65 | **-1.84*** | **-3.63, -0.05** | **-3.12*** | **-5.76, -0.49** |
| WIMD |  |  |  |  |  |  |  |  |
| *Q1 Most deprived 20% (ref)* |  |  | — | — | — | — | — | — |
| *Q2* |  |  | 0.67 | -0.02, 1.35 | 0.61 | -0.08, 1.30 | 0.50 | -0.27, 1.28 |
| *Q3* |  |  | **0.68*** | **0.01, 1.36** | 0.56 | -0.12, 1.25 | 0.03 | -0.78, 0.83 |
| *Q4* |  |  | **1.28***** | **0.59, 1.97** | **1.13**** | **0.43, 1.84** | **1.41**** | **0.55, 2.27** |
| *Q5 Least deprived 20%* |  |  | **1.41***** | **0.70, 2.12** | **1.36***** | **0.64, 2.07** | **1.41***** | **0.59, 2.23** |
| Gender |  |  |  |  |  |  |  |  |
| *Female (ref)* |  |  | — | — | — | — | — | — |
| *Male* |  |  | **0.70***** | **0.31, 1.10** | **0.72***** | **0.32, 1.11** | **0.66*** | **0.15, 1.17** |
| Age |  |  |  |  |  |  |  |  |
| *16-24* |  |  | — | — | — | — | — | — |
| *25-44* |  |  | -0.01 | -0.68, 0.67 | 0.02 | -0.66, 0.70 | **-1.27**** | **-2.11, -0.42** |
| *45-64* |  |  | -0.10 | -0.76, 0.56 | -0.10 | -0.76, 0.55 | **-1.32**** | **-2.15, -0.48** |
| *65-79* |  |  | **2.25***** | **1.51, 2.99** | **2.23***** | 1.49, 2.97 | 0.85 | -0.10, 1.79 |
| *80+* |  |  | 0.82 | -0.30, 1.95 | 0.82 | -0.30, 1.94 | -0.40 | -1.83, 1.03 |
| Economic status |  |  |  |  |  |  |  |  |
| *Employed (ref)* |  |  | — | — | — | — | — | — |
| *Inactive* |  |  | **-1.30***** | **-1.81, -0.79** | **-1.28***** | **-1.79, -0.77** | **-1.02**** | **-1.66, -0.37** |
| *Unemployed* |  |  | **-1.53*** | **-2.89, -0.16** | **-1.51*** | **-2.87, -0.14** | **-2.47**** | **-4.10, -0.85** |
| Material deprivation |  |  |  |  |  |  |  |  |
| *Not in material deprivation (ref)* |  |  | **—** | **—** | **—** | **—** | **—** | **—** |
| *In material deprivation* |  |  | **-6.26***** | **-6.89, -5.63** | **-6.28***** | **-6.91, -5.64** | **-6.33***** | **-7.12, -5.54** |
| Car use |  |  |  |  |  |  |  |  |
| *No (ref)* |  |  | — | — | — | — | — | — |
| *Yes* |  |  | **1.87***** | **1.17, 2.57** | **1.87***** | **1.17, 2.57** | **1.75***** | **0.93, 2.57** |
| Season |  |  |  |  |  |  |  |  |
| *Autumn (ref)* |  |  | — | — | — | — | — | — |
| *Spring* |  |  | -0.01 | -0.55, 0.53 | -0.05 | -0.59, 0.49 | 0.17 | -0.53, 0.86 |
| *Summer* |  |  | 0.26 | -0.30, 0.82 | 0.24 | -0.32, 0.80 | 0.50 | -0.22, 1.23 |
| *Winter* |  |  | -0.40 | -0.98, 0.18 | -0.41 | -1.00, 0.17 | -0.59 | -1.33, 0.16 |
| Wave |  |  |  |  |  |  |  |  |
| *2016-17 (ref)* |  |  | — | — | — | — | — | — |
| *2018-19* |  |  | **0.62**** | **0.21, 1.03** | **0.57**** | **0.15, 0.99** | **0.58*** | **0.05, 1.11** |
| Urban status |  |  |  |  |  |  |  |  |
| *Urban* |  |  | — | — | — | — | — | — |
| *Town & fringe* |  |  |  |  | 0.53 | -0.06, 1.12 |  |  |
| *Village, hamlet & isolated dwellings* |  |  |  |  | 0.54 | -0.02, 1.10 |  |  |
| AIC | 57,518 |  | 56,784 |  | 56,783 |  | 35,039 |  |
| No. Obs. | 7,631 |  | 7,631 |  | 7,631 |  | 4,702 |  |
| *R^2^* | 0.000 |  | 0.096 |  | 0.097 |  | 0.107 |  |
| ^1^**p*<0.05; ***p*<0.01; ****p*<0.001 | | | | | | | | |
| ^2^CI = Confidence Interval | | | | | | | | |

Table 2.3 Generalised linear model results predicting life satisfaction by EVI. Models include unadjusted with no covariates, adjusted with covariates, additionally adjusted with urban status, and adjusted for those residing in urban areas only.

|  | Life satisfaction | | | | | | | |
| --- | --- | --- | --- | --- | --- | --- | --- | --- |
|  | Unadjusted | | Adjusted | | Adj. with urban | | Adj., urban only | |
| Characteristic | Beta^1^ | 95% CI^2^ | Beta^1^ | 95% CI^2^ | Beta^1^ | 95% CI^2^ | Beta^1^ | 95% CI^2^ |
| Intercept | **7.73***** | **7.62, 7.84** | **7.90***** | **7.67, 8.13** | **7.96***** | **7.72, 8.19** | **7.87***** | **7.57, 8.17** |
| EVI | **0.32*** | **0.03, 0.61** | 0.07 | -0.23, 0.37 | -0.12 | -0.47, 0.22 | -0.21 | -0.72, 0.30 |
| WIMD |  |  |  |  |  |  |  |  |
| *Q1 Most deprived 20% (ref)* |  |  | — | — | — | — | — | — |
| *Q2* |  |  | 0.06 | -0.08, 0.19 | 0.05 | -0.08, 0.18 | 0.14 | -0.01, 0.29 |
| *Q3* |  |  | 0.12 | -0.01, 0.25 | 0.09 | -0.04, 0.23 | 0.05 | -0.11, 0.21 |
| *Q4* |  |  | 0.06 | -0.07, 0.19 | 0.03 | -0.10, 0.17 | 0.13 | -0.04, 0.29 |
| *Q5 Least deprived 20%* |  |  | **0.18*** | **0.04, 0.31** | **0.17*** | **0.03, 0.31** | **0.24**** | **0.08, 0.39** |
| Gender |  |  |  |  |  |  |  |  |
| *Female (ref)* |  |  | — | — | — | — | — | — |
| *Male* |  |  | -0.06 | -0.14, 0.01 | -0.06 | -0.14, 0.01 | **-0.11*** | **-0.21, -0.01** |
| Age |  |  |  |  |  |  |  |  |
| *16-24 (ref)* |  |  | — | — | — | — | — | — |
| *25-44* |  |  | -0.10 | -0.23, 0.03 | -0.10 | -0.23, 0.03 | -0.15 | -0.31, 0.01 |
| *45-64* |  |  | **-0.33***** | **-0.45, -0.20** | **-0.32***** | **-0.45, -0.20** | **-0.38***** | **-0.55, -0.22** |
| *65-79* |  |  | 0.12 | -0.02, 0.26 | 0.12 | -0.03, 0.26 | 0.00 | -0.18, 0.19 |
| *80+* |  |  | 0.06 | -0.15, 0.28 | 0.06 | -0.15, 0.28 | -0.08 | -0.36, 0.19 |
| Economic status |  |  |  |  |  |  |  |  |
| *Employed (ref)* |  |  | — | — | — | — | — | — |
| *Inactive* |  |  | **-0.25***** | **-0.34, -0.15** | **-0.24***** | **-0.34, -0.14** | **-0.14*** | **-0.26, -0.01** |
| *Unemployed* |  |  | **-0.54***** | **-0.80, -0.28** | **-0.53***** | **-0.79, -0.27** | **-0.51**** | **-0.83, -0.20** |
| Material deprivation |  |  |  |  |  |  |  |  |
| *Not in material deprivation (ref)* |  |  | — | — | — | — | — | — |
| *In material deprivation* |  |  | **-1.33***** | **-1.45, -1.21** | **-1.33***** | **-1.45, -1.21** | **-1.39***** | **-1.55, -1.24** |
| Car use |  |  |  |  |  |  |  |  |
| *No (ref)* |  |  | **—** | **—** | **—** | **—** | **—** | **—** |
| *Yes* |  |  | **0.33***** | **0.20, 0.47** | **0.33***** | **0.20, 0.47** | **0.42***** | **0.26, 0.58** |
| Season |  |  |  |  |  |  |  |  |
| *Autumn (ref)* |  |  | — | — | — | — | — | — |
| *Spring* |  |  | **-0.12*** | **-0.23, -0.02** | **-0.13*** | **-0.23, -0.03** | -0.09 | -0.23, 0.04 |
| *Summer* |  |  | **-0.14*** | **-0.25, -0.03** | **-0.14*** | **-0.25, -0.03** | -0.07 | -0.21, 0.07 |
| *Winter* |  |  | -0.05 | -0.17, 0.06 | -0.06 | -0.17, 0.05 | -0.04 | -0.19, 0.10 |
| Wave |  |  |  |  |  |  |  |  |
| *2016-17 (ref)* |  |  | — | — | — | — | — | — |
| *2018-19* |  |  | **0.09*** | **0.01, 0.17** | 0.08 | 0.00, 0.16 | **0.10*** | **0.00, 0.20** |
| Urban status |  |  |  |  |  |  |  |  |
| *Urban (ref)* |  |  | — | — | — | — | — | — |
| *Town & fringe* |  |  |  |  | 0.04 | -0.08, 0.15 |  |  |
| *Village, hamlet & isolated dwellings* | |  |  |  | **0.13*** | **0.02, 0.24** |  |  |
| AIC | 32,439 |  | 31,685 |  | 31,684 |  | 19,582 |  |
| No. Obs. | 7,631 |  | 7,631 |  | 7,631 |  | 4,702 |  |
| *R_2_* | 0.001 |  | 0.099 |  | 0.099 |  | 0.115 |  |
| ^1^**p*<0.05; ***p*<0.01; ****p*<0.001 | | | | | | | | |
| ^2^CI = Confidence Interval | | | | | | | | |

Table 2 4 Generalised linear model results predicting WEMWBS by proximity to nearest green or blue space (GBS). Models include unadjusted with no covariates, adjusted with covariates, additionally adjusted with urban status, additionally adjusted with local authority, and adjusted for those residing in urban areas only.

|  | WEMWBS | | | | | | | | | |
| --- | --- | --- | --- | --- | --- | --- | --- | --- | --- | --- |
|  | Unadjusted | | Adjusted | | Adj. with urban | | Adj. with LA | | Adj,. urban only | |
| Characteristic | Beta^1^ | 95% CI^2^ | Beta^1^ | 95% CI^2^ | Beta^1^ | 95% CI^2^ | Beta^1^ | 95% CI^2^ | Beta^1^ | 95% CI^2^ |
| Intercept | **51.15***** | **50.77, 51.53** | **49.21***** | **48.09, 50.34** | **49.18***** | **48.05, 50.31** | **50.70***** | **49.39, 52.00** | **51.90***** | **50.39, 53.42** |
| Proximity to nearest GBS |  |  |  |  |  |  |  |  |  |  |
| *0 - <100 (ref)* | — | — | — | — | — | — | — | — | — | — |
| *100 - <300* | 0.04 | -0.43, 0.52 | -0.16 | -0.61, 0.29 | -0.16 | -0.61, 0.30 | -0.21 | -0.67, 0.24 | **-0.60*** | **-1.17, -0.02** |
| *300 - <500* | -0.13 | -0.77, 0.52 | -0.52 | -1.14, 0.10 | -0.55 | -1.18, 0.07 | **-0.69*** | **-1.32, -0.06** | -0.42 | -1.29, 0.45 |
| *500 - 1100* | **1.46**** | **0.36, 2.57** | 0.46 | -0.60, 1.52 | 0.42 | -0.65, 1.48 | 0.21 | -0.87, 1.28 | 0.71 | -0.97, 2.39 |
| WIMD |  |  |  |  |  |  |  |  |  |  |
| *Q1 Most deprived 20% (ref)* | — | — | — | — | — | — | — | — | — | — |
| *Q2* |  |  | 0.64 | -0.05, 1.32 | 0.59 | -0.10, 1.28 | 0.62 | -0.08, 1.31 | 0.61 | -0.17, 1.39 |
| *Q3* |  |  | 0.62 | -0.04, 1.29 | 0.52 | -0.16, 1.21 | 0.42 | -0.28, 1.12 | 0.10 | -0.72, 0.92 |
| *Q4* |  |  | **1.20***** | **0.52, 1.88** | **1.08**** | **0.37, 1.78** | **1.04**** | **0.31, 1.77** | **1.34**** | **0.46, 2.23** |
| *Q5 Least deprived 20%* |  |  | **1.41***** | **0.70, 2.12** | **1.36***** | **0.65, 2.08** | **1.01**** | **0.27, 1.75** | **0.93*** | **0.08, 1.78** |
| Gender |  |  |  |  |  |  |  |  |  |  |
| *Female (ref)* | — | — | — | — | — | — | — | — | — | — |
| *Male* |  |  | **0.70***** | **0.30, 1.09** | **0.71***** | **0.31, 1.10** | **0.74***** | **0.34, 1.13** | **0.67**** | **0.16, 1.17** |
| Age |  |  |  |  |  |  |  |  |  |  |
| 16-24 (ref) | — | — | — | — | — | — | — | — | — | — |
| 25-44 |  |  | 0.01 | -0.67, 0.69 | 0.02 | -0.66, 0.70 | 0.00 | -0.68, 0.68 | **-1.28**** | **-2.12, -0.43** |
| 45-64 |  |  | -0.10 | -0.76, 0.56 | -0.11 | -0.77, 0.54 | -0.14 | -0.80, 0.51 | **-1.32**** | **-2.15, -0.48** |
| 65-79 |  |  | **2.22***** | **1.48, 2.95** | **2.19***** | **1.45, 2.92** | **2.16***** | **1.42, 2.90** | 0.84 | -0.11, 1.78 |
| 80+ |  |  | 0.82 | -0.31, 1.94 | 0.80 | -0.32, 1.93 | 0.86 | -0.26, 1.98 | -0.23 | -1.66, 1.20 |
| Economic status |  |  |  |  |  |  |  |  |  |  |
| *Employed (ref)* | — | — | — | — | — | — | — | — | — | — |
| *Inactive* |  |  | **-1.29***** | **-1.80, -0.78** | **-1.27***** | **-1.78, -0.76** | **-1.27***** | **-1.78, -0.76** | **-1.05**** | **-1.70, -0.41** |
| *Unemployed* |  |  | **-1.50*** | **-2.86, -0.13** | **-1.49*** | **-2.86, -0.13** | -1.36 | -2.72, 0.01 | **-2.31**** | **-3.93, -0.68** |
| Material deprivation |  |  |  |  |  |  |  |  |  |  |
| *Not in material deprivation (ref)* | | — | — | — | — | — | — | — | — | — |
| *In material deprivation* |  |  | **-6.27***** | **-6.90, -5.64** | **-6.28***** | **-6.92, -5.65** | **-6.32***** | **-6.95, -5.69** | **-6.39***** | **-7.19, -5.60** |
| Car use |  |  |  |  |  |  |  |  |  |  |
| *No (ref)* | — | — | **—** | **—** | **—** | **—** | **—** | **—** | **—** | **—** |
| *Yes* |  |  | **1.82***** | **1.12, 2.52** | **1.80***** | **1.10, 2.50** | **1.89***** | **1.19, 2.59** | **1.75***** | **0.93, 2.57** |
| Season |  |  |  |  |  |  |  |  |  |  |
| *Autumn (ref)* | — | — | — | — | — | — | — | — | — | — |
| *Spring* |  |  | 0.01 | -0.53, 0.55 | 0.00 | -0.54, 0.54 | -0.10 | -0.64, 0.44 | 0.06 | -0.63, 0.76 |
| *Summer* |  |  | 0.27 | -0.29, 0.83 | 0.26 | -0.30, 0.82 | 0.21 | -0.35, 0.77 | 0.39 | -0.33, 1.12 |
| *Winter* |  |  | -0.39 | -0.97, 0.19 | -0.39 | -0.97, 0.20 | -0.47 | -1.06, 0.11 | **-0.75*** | **-1.50, -0.01** |
| Wave |  |  |  |  |  |  |  |  |  |  |
| *2016-17 (ref)* | — | — | — | — | — | — | — | — | — | — |
| *2018-19* |  |  | **0.68***** | **0.28, 1.08** | **0.69***** | **0.30, 1.09** | **0.71***** | **0.31, 1.11** | **0.76**** | **0.25, 1.27** |
| Urban status |  |  |  |  |  |  |  |  |  |  |
| *Urban* | — | — | — | — | — | — | — | — | — | — |
| *Town & fringe* |  |  |  |  | 0.40 | -0.18, 0.97 | 0.16 | -0.47, 0.79 |  |  |
| *Village, hamlet & isolated dwellings* | |  |  |  | 0.27 | -0.23, 0.77 | 0.13 | -0.45, 0.70 |  |  |
| Local authority |  |  |  |  |  |  |  |  |  |  |
| *Cardiff* | — | — | — | — | — | — | — | — | — | — |
| *Blaenau Gwent* |  |  |  |  |  |  | **-2.31***** | **-3.60, -1.02** | **-1.98**** | **-3.31, -0.66** |
| *Bridgend* |  |  |  |  |  |  | -0.30 | -1.57, 0.96 | 0.01 | -1.43, 1.45 |
| *Caerphilly* |  |  |  |  |  |  | **-2.43***** | **-3.54, -1.33** | **-1.98**** | **-3.18, -0.78** |
| *Carmarthenshire* |  |  |  |  |  |  | **-2.43***** | **-3.58, -1.28** | **-2.07**** | **-3.50, -0.63** |
| *Ceredigion* |  |  |  |  |  |  | -0.53 | -1.96, 0.89 | 0.50 | -2.36, 3.35 |
| *Conwy* |  |  |  |  |  |  | -0.33 | -1.59, 0.93 | -0.34 | -1.84, 1.15 |
| *Denbighshire* |  |  |  |  |  |  | -0.22 | -1.47, 1.03 | 0.13 | -1.53, 1.80 |
| *Flintshire* |  |  |  |  |  |  | -0.28 | -1.40, 0.83 | 0.58 | -0.66, 1.82 |
| *Gwynedd* |  |  |  |  |  |  | -0.44 | -1.81, 0.92 | 0.20 | -3.91, 4.31 |
| *Isle of Anglesey* |  |  |  |  |  |  | 0.11 | -1.35, 1.57 | 1.81 | -1.92, 5.54 |
| *Merthyr Tydfil* |  |  |  |  |  |  | -0.90 | -2.55, 0.74 | -0.87 | -2.59, 0.86 |
| *Monmouthshire* |  |  |  |  |  |  | **-1.30*** | **-2.46, -0.14** | **-1.60*** | **-3.01, -0.18** |
| *Neath Port Talbot* |  |  |  |  |  |  | **-1.96**** | **-3.27, -0.64** | **-2.21**** | **-3.67, -0.74** |
| *Newport* |  |  |  |  |  |  | **-2.22***** | **-3.34, -1.11** | **-1.99***** | **-3.16, -0.83** |
| *Pembrokeshire* |  |  |  |  |  |  | **-1.67**** | **-2.92, -0.41** | **-3.06**** | **-5.28, -0.85** |
| *Powys* |  |  |  |  |  |  | **-1.51**** | **-2.57, -0.44** | **-2.53*** | **-4.52, -0.53** |
| *Rhondda Cynon Taf* |  |  |  |  |  |  | **-1.52*** | **-2.68, -0.36** | **-2.06**** | **-3.29, -0.82** |
| *Swansea* |  |  |  |  |  |  | **-1.25*** | -2.28, -0.21 | -1.02 | -2.11, 0.07 |
| *Torfaen* |  |  |  |  |  |  | **-3.30***** | -4.47, -2.12 | **-3.67***** | **-4.91, -2.43** |
| *Vale of Glamorgan* |  |  |  |  |  |  | **-1.60*** | -2.83, -0.37 | **-1.48*** | **-2.80, -0.16** |
| *Wrexham* |  |  |  |  |  |  | **-1.50*** | -2.79, -0.22 | -1.00 | -2.53, 0.54 |
| AIC | 57,516 |  | 56,785 |  | 56,787 |  | 56,748 |  | 35,006 |  |
| No. Obs. | 7,631 |  | 7,631 |  | 7,631 |  | 7,631 |  | 4,702 |  |
| *R^2^* | 0.001 |  | 0.096 |  | 0.097 |  | 0.106 |  | 0.122 |  |
| ^1^**p*<0.05; ***p*<0.01; ****p*<0.001 | | | | | | | | | | |
| ^2^CI = Confidence Interval | | | | | | | | | | |

Table 2.5 Generalised linear model results predicting life satisfaction by proximity to nearest green or blue space (GBS). Models include unadjusted with no covariates, adjusted with covariates, additionally adjusted with urban status, additionally adjusted with local authority, and adjusted for those residing in urban areas only.

|  | Life satisfaction | | | | | | | | | |
| --- | --- | --- | --- | --- | --- | --- | --- | --- | --- | --- |
|  | Unadjusted | | Adjusted | | Adj. with urban | | Adj. with LA | | Adj,. urban only | |
| Characteristic | Beta^1^ | 95% CI^2^ | Beta^1^ | 95% CI^2^ | Beta^1^ | 95% CI^2^ | Beta^1^ | 95% CI^2^ | Beta^1^ | 95% CI^2^ |
| Intercept | **7.85***** | **7.77, 7.92** | **7.96***** | **7.74, 8.17** | **7.95***** | **7.73, 8.17** | **7.98***** | **7.73, 8.23** | **7.91***** | **7.62, 8.20** |
| Proximity to nearest GBS (m) |  |  |  |  |  |  |  |  |  |  |
| *0 - <100* | — | — | — | — | — | — | — | — | — | — |
| *100 - <300* | -0.01 | -0.10, 0.08 | -0.04 | -0.13, 0.04 | -0.04 | -0.13, 0.05 | -0.04 | -0.13, 0.04 | -0.07 | -0.18, 0.04 |
| *300 - <500* | -0.01 | -0.13, 0.12 | -0.07 | -0.19, 0.05 | -0.08 | -0.20, 0.04 | -0.08 | -0.21, 0.04 | -0.10 | -0.27, 0.07 |
| *500 - 1100* | 0.16 | -0.06, 0.37 | -0.01 | -0.22, 0.19 | -0.02 | -0.23, 0.18 | -0.05 | -0.26, 0.15 | 0.25 | -0.07, 0.58 |
| WIMD |  |  |  |  |  |  |  |  |  |  |
| *Q1 Most deprived 20% (ref)* | — | — | — | — | — | — | — | — | — | — |
| *Q2* |  |  | 0.06 | -0.07, 0.19 | 0.05 | -0.08, 0.18 | 0.05 | -0.09, 0.18 | 0.12 | -0.04, 0.27 |
| *Q3* |  |  | 0.12 | -0.01, 0.25 | 0.09 | -0.04, 0.23 | 0.08 | -0.05, 0.22 | 0.03 | -0.13, 0.19 |
| *Q4* |  |  | 0.07 | -0.06, 0.20 | 0.03 | -0.10, 0.17 | 0.06 | -0.08, 0.20 | 0.10 | -0.07, 0.27 |
| *Q5 Least deprived 20%* |  |  | **0.19**** | **0.05, 0.32** | **0.18*** | **0.04, 0.31** | **0.20**** | **0.06, 0.35** | **0.21*** | **0.04, 0.37** |
| Gender |  |  |  |  |  |  |  |  |  |  |
| *Female (ref)* | — | — | — | — | — | — | — | — | — | — |
| *Male* |  |  | -0.07 | -0.14, 0.01 | -0.06 | -0.14, 0.01 | -0.06 | -0.13, 0.02 | **-0.11*** | **-0.21, -0.01** |
| Age |  |  |  |  |  |  |  |  |  |  |
| *16-24 (ref)* | — | — | — | — | — | — | — | — | — | — |
| *25-44* |  |  | -0.10 | -0.23, 0.03 | -0.10 | -0.23, 0.04 | -0.10 | -0.23, 0.04 | -0.15 | -0.31, 0.02 |
| *45-64* |  |  | **-0.32***** | **-0.45, -0.20** | **-0.32***** | **-0.45, -0.20** | **-0.33***** | **-0.46, -0.21** | **-0.38***** | **-0.54, -0.22** |
| *65-79* |  |  | 0.12 | -0.02, 0.27 | 0.12 | -0.03, 0.26 | 0.11 | -0.03, 0.25 | 0.01 | -0.17, 0.20 |
| *80+* |  |  | 0.07 | -0.15, 0.29 | 0.07 | -0.15, 0.28 | 0.07 | -0.15, 0.28 | -0.07 | -0.35, 0.21 |
| Economic status |  |  |  |  |  |  |  |  |  |  |
| *Employed (ref)* | — | — | — | — | — | — | — | — | — | — |
| *Inactive* |  |  | **-0.25***** | **-0.34, -0.15** | **-0.24***** | **-0.34, -0.14** | **-0.25***** | **-0.35, -0.15** | **-0.15*** | **-0.28, -0.03** |
| *Unemployed* |  |  | **-0.53***** | **-0.80, -0.27** | **-0.53***** | **-0.79, -0.26** | **-0.52***** | **-0.79, -0.26** | **-0.50**** | **-0.82, -0.19** |
| Material deprivation |  |  |  |  |  |  |  |  |  |  |
| *Not in material deprivation (ref)* | | — | — | — | — | — | — | — | — | — |
| *In material deprivation* |  |  | **-1.33***** | **-1.45, -1.21** | **-1.33***** | **-1.45, -1.21** | **-1.33***** | **-1.45, -1.21** | **-1.39***** | **-1.54, -1.23** |
| Car use |  |  |  |  |  |  |  |  |  |  |
| *No (ref)* | — | — | — | — | — | — | — | — | — | — |
| *Yes* |  |  | **0.34***** | **0.20, 0.47** | **0.33***** | **0.19, 0.46** | **0.33***** | **0.20, 0.47** | **0.42***** | **0.26, 0.58** |
| Season |  |  |  |  |  |  |  |  |  |  |
| *Autumn (ref)* | — | — | — | — | — | — | — | — | — | — |
| *Spring* |  |  | **-0.12*** | **-0.23, -0.02** | **-0.12*** | **-0.23, -0.02** | **-0.13*** | **-0.24, -0.03** | -0.11 | -0.25, 0.02 |
| *Summer* |  |  | **-0.14*** | **-0.25, -0.03** | **-0.14*** | **-0.25, -0.03** | **-0.14*** | **-0.25, -0.03** | -0.07 | -0.21, 0.07 |
| *Winter* |  |  | -0.05 | -0.17, 0.06 | -0.06 | -0.17, 0.06 | -0.06 | -0.17, 0.05 | -0.06 | -0.20, 0.09 |
| Wave |  |  |  |  |  |  |  |  |  |  |
| *2016-17 (ref)* | — | — | — | — | — | — | — | — | — | — |
| *2018-19* |  |  | **0.09*** | **0.01, 0.16** | **0.08*** | **0.01, 0.16** | 0.08 | 0.00, 0.15 | **0.10*** | **0.00, 0.20** |
| Urban status |  |  |  |  |  |  |  |  |  |  |
| *Urban* | — | — | — | — | — | — | — | — | — | — |
| *Town & fringe* |  |  |  |  | 0.03 | -0.08, 0.14 | -0.02 | -0.14, 0.11 |  |  |
| *Village, hamlet & isolated dwellings* | |  |  |  | **0.11*** | **0.02, 0.21** | 0.08 | -0.03, 0.19 |  |  |
| Local authority |  |  |  |  |  |  |  |  |  |  |
| *Cardiff* | — | — | — | — | — | — | — | — | — | — |
| *Blaenau Gwent* |  |  |  |  |  |  | -0.03 | -0.28, 0.22 | -0.03 | -0.29, 0.23 |
| *Bridgend* |  |  |  |  |  |  | **0.27*** | **0.03, 0.51** | **0.32*** | **0.05, 0.60** |
| *Caerphilly* |  |  |  |  |  |  | 0.08 | -0.14, 0.29 | 0.13 | -0.10, 0.37 |
| *Carmarthenshire* |  |  |  |  |  |  | -0.15 | -0.38, 0.07 | -0.14 | -0.42, 0.14 |
| *Ceredigion* |  |  |  |  |  |  | 0.25 | -0.03, 0.53 | 0.31 | -0.25, 0.86 |
| *Conwy* |  |  |  |  |  |  | 0.00 | -0.24, 0.24 | 0.03 | -0.26, 0.32 |
| *Denbighshire* |  |  |  |  |  |  | 0.10 | -0.14, 0.35 | 0.13 | -0.20, 0.45 |
| *Flintshire* |  |  |  |  |  |  | -0.02 | -0.24, 0.19 | 0.01 | -0.23, 0.25 |
| *Gwynedd* |  |  |  |  |  |  | 0.19 | -0.08, 0.45 | 0.14 | -0.66, 0.93 |
| *Isle of Anglesey* |  |  |  |  |  |  | 0.19 | -0.09, 0.48 | -0.31 | -1.04, 0.41 |
| *Merthyr Tydfil* |  |  |  |  |  |  | -0.03 | -0.35, 0.28 | -0.05 | -0.38, 0.29 |
| *Monmouthshire* |  |  |  |  |  |  | 0.08 | -0.14, 0.31 | 0.06 | -0.21, 0.34 |
| *Neath Port Talbot* |  |  |  |  |  |  | 0.04 | -0.22, 0.29 | 0.03 | -0.26, 0.31 |
| *Newport* |  |  |  |  |  |  | -0.12 | -0.34, 0.09 | -0.10 | -0.33, 0.13 |
| *Pembrokeshire* |  |  |  |  |  |  | 0.03 | -0.21, 0.27 | -0.37 | -0.80, 0.06 |
| *Powys* |  |  |  |  |  |  | -0.14 | -0.34, 0.07 | 0.08 | -0.31, 0.47 |
| *Rhondda Cynon Taf* |  |  |  |  |  |  | -0.10 | -0.32, 0.12 | -0.13 | -0.37, 0.11 |
| *Swansea* |  |  |  |  |  |  | -0.15 | -0.35, 0.05 | -0.17 | -0.38, 0.05 |
| *Torfaen* |  |  |  |  |  |  | 0.00 | -0.23, 0.22 | -0.02 | -0.26, 0.22 |
| *Vale of Glamorgan* |  |  |  |  |  |  | **-0.24*** | **-0.48, 0.00** | -0.14 | -0.39, 0.12 |
| *Wrexham* |  |  |  |  |  |  | -0.08 | -0.33, 0.17 | -0.04 | -0.34, 0.26 |
| AIC | 32,445 |  | 31,688 |  | 31,687 |  | 31,687 |  | 19,596 |  |
| No. Obs. | 7,631 |  | 7,631 |  | 7,631 |  | 7,631 |  | 4,702 |  |
| *R_2_* | 0.000 |  | 0.099 |  | 0.099 |  | 0.104 |  | 0.121 |  |
| ^1^**p*<0.05; ***p*<0.01; ****p*<0.001 | | | | | | | | | | |
| ^2^CI = Confidence Interval | | | | | | | | | | |

Table 2.6 Generalised linear model results predicting WEMWBS by weekly leisure time outdoors. Models include unadjusted with no covariates, adjusted with covariates, additionally adjusted with urban status, and adjusted for those residing in urban areas only.

|  | WEMWBS | | | | | | | |
| --- | --- | --- | --- | --- | --- | --- | --- | --- |
|  | Unadjusted | | Adjusted | | Adj with urban | | Adj., urban only | |
| Characteristic | Beta^1^ | 95% CI^2^ | Beta^1^ | 95% CI^2^ | Beta^1^ | 95% CI^2^ | Beta^1^ | 95% CI^2^ |
| Intercept | **48.89***** | **48.50, 49.29** | **47.45***** | **46.32, 48.59** | **47.43***** | **46.29, 48.56** | **48.54***** | **47.16, 49.91** |
| Weekly time outdoors (mins) |  |  |  |  |  |  |  |  |
| *0 (ref)* | — | — | — | — | — | — | — | — |
| *>0 - <60* | **2.41***** | **1.77, 3.05** | **2.02***** | **1.39, 2.65** | **2.02***** | **1.39, 2.65** | **1.81***** | **1.01, 2.60** |
| *60 - <120* | **2.41***** | **1.73, 3.08** | **1.89***** | **1.22, 2.57** | **1.90***** | **1.23, 2.57** | **2.35***** | **1.51, 3.20** |
| *120 - <240* | **2.79***** | **2.16, 3.42** | **2.36***** | **1.74, 2.99** | **2.36***** | **1.74, 2.98** | **2.28***** | **1.46, 3.09** |
| *240 - 420* | **4.17***** | **3.61, 4.72** | **3.58***** | **3.03, 4.13** | **3.57***** | **3.02, 4.13** | **3.23***** | **2.50, 3.96** |
| WIMD |  |  |  |  |  |  |  |  |
| *Q1 Most deprived 20% (ref)* | — | — | — | — | — | — | — | — |
| *Q2* |  |  | 0.50 | -0.18, 1.18 | 0.47 | -0.22, 1.15 | 0.34 | -0.43, 1.11 |
| *Q3* |  |  | 0.41 | -0.26, 1.07 | 0.34 | -0.33, 1.02 | -0.13 | -0.94, 0.67 |
| *Q4* |  |  | **0.98**** | **0.31, 1.65** | **0.90*** | **0.20, 1.59** | **1.21**** | **0.35, 2.06** |
| *Q5 Least deprived 20%* |  |  | **1.08**** | **0.38, 1.78** | **1.05**** | **0.34, 1.75** | **0.98*** | **0.17, 1.79** |
| Gender |  |  |  |  |  |  |  |  |
| *Female (ref)* | — | — | — | — | — | — | — | — |
| *Male* |  |  | **0.67***** | **0.28, 1.06** | **0.67***** | **0.28, 1.07** | **0.64*** | **0.14, 1.14** |
| Age |  |  |  |  |  |  |  |  |
| *16-24 (ref)* | — | — | — | — | — | — | — | — |
| *25-44* |  |  | -0.08 | -0.75, 0.59 | -0.07 | -0.75, 0.60 | **-1.33**** | **-2.17, -0.49** |
| *45-64* |  |  | 0.11 | -0.55, 0.76 | 0.10 | -0.56, 0.75 | **-1.02*** | **-1.85, -0.18** |
| *65-79* |  |  | **2.63***** | **1.89, 3.37** | **2.61***** | **1.87, 3.35** | **1.30**** | **0.36, 2.25** |
| *80+* |  |  | **1.86**** | **0.73, 3.00** | **1.86**** | **0.72, 2.99** | 0.64 | -0.80, 2.08 |
| Economic status |  |  |  |  |  |  |  |  |
| *Employed (ref)* | — | — | — | — | — | — | — | — |
| *Inactive* |  |  | **-1.13***** | **-1.63, -0.62** | **-1.12***** | **-1.62, -0.61** | **-0.89**** | **-1.54, -0.25** |
| *Unemployed* |  |  | **-1.40*** | **-2.76, -0.05** | **-1.40*** | **-2.76, -0.05** | **-2.34**** | **-3.95, -0.73** |
| Material deprivation |  |  |  |  |  |  |  |  |
| *Not in material deprivation (ref)* | — | — | — | — | — | — | — | — |
| *In material deprivation* |  |  | **-5.99***** | **-6.62, -5.37** | **-6.00***** | **-6.63, -5.37** | **-6.02***** | **-6.81, -5.23** |
| Car use |  |  |  |  |  |  |  |  |
| *No (ref)* | — | — | — | — | — | — | — | — |
| *Yes* |  |  | **1.41***** | **0.72, 2.11** | **1.40***** | **0.70, 2.09** | **1.31**** | **0.49, 2.13** |
| Season |  |  |  |  |  |  |  |  |
| *Autumn (ref)* | — | — | — | — | — | — | — | — |
| *Spring* |  |  | 0.11 | -0.43, 0.64 | 0.10 | -0.44, 0.63 | 0.34 | -0.34, 1.03 |
| *Summer* |  |  | 0.20 | -0.35, 0.76 | 0.20 | -0.36, 0.76 | 0.42 | -0.29, 1.14 |
| *Winter* |  |  | -0.18 | -0.76, 0.40 | -0.18 | -0.76, 0.40 | -0.36 | -1.10, 0.38 |
| Wave |  |  |  |  |  |  |  |  |
| *2016-17 (ref)* | — | — | — | — | — | — | — | — |
| *2018-19* |  |  | **0.48*** | **0.08, 0.87** | **0.49*** | **0.09, 0.88** | **0.57*** | **0.07, 1.08** |
| Urban status |  |  |  |  |  |  |  |  |
| *Urban (ref)* | — | — | — | — | — | — | — | — |
| *Town & fringe* |  |  |  |  | 0.27 | -0.30, 0.84 |  |  |
| *Village, hamlet & isolated dwellings* | |  |  |  | 0.16 | -0.33, 0.65 |  |  |
| AIC | 57,306 |  | 56,630 |  | 56,633 |  | 34,971 |  |
| No. Obs. | 7,631 |  | 7,631 |  | 7,631 |  | 4,702 |  |
| *R^2^* | 0.028 |  | 0.115 |  | 0.115 |  | 0.121 |  |
| ^1^*p<0.05; ***p*<0.01; ****p*<0.001 | | | | | | | | |
| ^2^CI = Confidence Interval | | | | | | | | |

Table 2 7 Generalised linear model results predicting life satisfaction by weekly leisure time outdoors. Models include unadjusted with no covariates, adjusted with covariates, additionally adjusted with urban status, and adjusted for those residing in urban areas only.

|  | Life satisfaction | | | | | | | |
| --- | --- | --- | --- | --- | --- | --- | --- | --- |
|  | Unadj | | Adj | | Adj with urban | | Adj urb only | |
| Characteristic | Beta^1^ | 95% CI^2^ | Beta^1^ | 95% CI^2^ | Beta^1^ | 95% CI^2^ | Beta^1^ | 95% CI^2^ |
| Intercept | **7.46***** | **7.39, 7.54** | **7.68***** | **7.46, 7.90** | **7.68***** | **7.46, 7.90** | **7.58***** | **7.32, 7.85** |
| Weekly time outdoors (mins) |  |  |  |  |  |  |  |  |
| *0 (ref)* | **—** | **—** | **—** | **—** | **—** | **—** | **—** | **—** |
| *>0 - <60* | **0.52***** | **0.40, 0.64** | **0.39***** | **0.27, 0.51** | **0.39***** | **0.27, 0.51** | **0.33***** | **0.17, 0.48** |
| *60 - <120* | **0.42***** | **0.29, 0.56** | **0.25***** | **0.12, 0.38** | **0.25***** | **0.12, 0.38** | **0.33***** | **0.16, 0.49** |
| *120 - <240* | **0.43***** | **0.31, 0.55** | **0.31***** | **0.19, 0.43** | **0.31***** | **0.19, 0.43** | **0.22**** | **0.06, 0.37** |
| *240 - 420* | **0.62***** | **0.51, 0.73** | **0.49***** | **0.38, 0.60** | **0.49***** | **0.38, 0.59** | **0.42***** | **0.28, 0.56** |
| WIMD |  |  |  |  |  |  |  |  |
| *Q1 Most deprived 20% (ref)* | — | — | — | — | — | — | — | — |
| *Q2* |  |  | 0.04 | -0.09, 0.17 | 0.03 | -0.10, 0.17 | 0.12 | -0.02, 0.27 |
| *Q3* |  |  | 0.10 | -0.03, 0.23 | 0.07 | -0.06, 0.20 | 0.04 | -0.12, 0.19 |
| *Q4* |  |  | 0.04 | -0.09, 0.17 | 0.01 | -0.13, 0.14 | 0.11 | -0.06, 0.27 |
| *Q5 Least deprived 20%* |  |  | **0.14*** | **0.00, 0.28** | 0.13 | -0.01, 0.27 | **0.19*** | **0.03, 0.35** |
| Gender |  |  |  |  |  |  |  |  |
| *Female (ref)* | — | — | — | — | — | — | — | — |
| *Male* |  |  | -0.07 | -0.14, 0.01 | -0.06 | -0.14, 0.01 | **-0.11*** | **-0.20, -0.01** |
| Age |  |  |  |  |  |  |  |  |
| *16-24 (ref)* | — | — | — | — | — | — | — | — |
| *25-44* |  |  | -0.12 | -0.25, 0.02 | -0.11 | -0.24, 0.02 | -0.16 | -0.32, 0.00 |
| *45-64* |  |  | **-0.29***** | **-0.42, -0.17** | **-0.29***** | **-0.42, -0.17** | **-0.34***** | **-0.50, -0.18** |
| *65-79* |  |  | **0.18*** | **0.04, 0.33** | **0.18*** | **0.03, 0.32** | 0.07 | -0.12, 0.25 |
| *80+* |  |  | 0.22 | 0.00, 0.44 | 0.21 | -0.01, 0.43 | 0.06 | -0.22, 0.34 |
| Economic status |  |  |  |  |  |  |  |  |
| *Employed (ref)* | — | — | — | — | — | — | — | — |
| *Inactive* |  |  | **-0.22***** | **-0.32, -0.12** | **-0.22***** | **-0.32, -0.12** | -0.12 | -0.24, 0.01 |
| *Unemployed* |  |  | **-0.51***** | **-0.78, -0.25** | **-0.51***** | **-0.77, -0.25** | **-0.50**** | **-0.81, -0.18** |
| Material deprivation |  |  |  |  |  |  |  |  |
| *Not in material deprivation (ref)* | — | — | — | — | — | — | — | — |
| *In material deprivation* |  |  | **-1.29***** | **-1.41, -1.17** | **-1.29***** | **-1.41, -1.17** | **-1.35***** | **-1.50, -1.20** |
| Car use |  |  |  |  |  |  |  |  |
| *No (ref)* | — | — | — | — | — | — | — | — |
| *Yes* |  |  | **0.28***** | **0.14, 0.41** | **0.27***** | **0.14, 0.41** | **0.37***** | **0.21, 0.53** |
| Season |  |  |  |  |  |  |  |  |
| *Autumn (ref)* | — | — | — | — | — | — | — | — |
| *Spring* |  |  | **-0.11*** | **-0.21, 0.00** | **-0.11*** | **-0.21, -0.01** | -0.08 | -0.21, 0.06 |
| *Summer* |  |  | **-0.14**** | **-0.25, -0.04** | **-0.15**** | **-0.25, -0.04** | -0.08 | -0.22, 0.06 |
| *Winter* |  |  | -0.02 | -0.14, 0.09 | -0.03 | -0.14, 0.09 | -0.02 | -0.16, 0.12 |
| Wave |  |  |  |  |  |  |  |  |
| *2016-17 (ref)* | — | — | — | — | — | — | — | — |
| *2018-19* |  |  | 0.06 | -0.02, 0.13 | 0.06 | -0.02, 0.13 | 0.09 | -0.01, 0.19 |
| Urban status |  |  |  |  |  |  |  |  |
| *Urban (ref)* | — | — | — | — | — | — | — | — |
| *Town & fringe* |  |  |  |  | 0.02 | -0.09, 0.13 |  |  |
| *Village, hamlet & isolated dwellings* | |  |  |  | 0.10* | 0.00, 0.19 |  |  |
| AIC | 32,309 |  | 31,606 |  | 31,605 |  | 19,551 |  |
| No. Obs. | 7,631 |  | 7,631 |  | 7,631 |  | 4,702 |  |
| *^R2^* | 0.018 |  | 0.109 |  | 0.109 |  | 0.122 |  |
| ^1^**p*<0.05; ***p*<0.01; ****p*<0.001 | | | | | | | | |
| ^2^CI = Confidence Interval | | | | | | | | |

Table 2.8 Generalised linear model results predicting subjective well-being (WEMWBS and life satisfaction) by environmental exposures (EVI, proximity to nearest GBS from the home, and weekly leisure time outdoors), including an interaction between the environmental exposure and material deprivation. Models are fully adjusted.

|  | EVI | | | | Proximity to nearest GBS | | | | Time outdoors | | | |
| --- | --- | --- | --- | --- | --- | --- | --- | --- | --- | --- | --- | --- |
|  | WEMWBS | | Life satisfaction | | WEMWBS | | Life satisfaction | | WEMWBS | | Life satisfaction | |
| Characteristic | Beta^1^ | 95% CI^2^ | Beta^1^ | 95% CI^2^ | Beta^1^ | 95% CI^2^ | Beta^1^ | 95% CI^2^ | Beta^1^ | 95% CI^2^ | Beta^1^ | 95% CI^2^ |
| Intercept | **49.48***** | **48.25, 50.72** | **7.99***** | **7.76, 8.23** | **50.74***** | **49.43, 52.05** | **7.99***** | **7.74, 8.24** | **47.73***** | **46.58, 48.88** | **7.74***** | **7.52, 7.96** |
| EVI | -1.61 | -3.48, 0.26 | -0.21 | -0.57, 0.15 |  |  |  |  |  |  |  |  |
| Proximity to nearest GBS | |  |  |  |  |  |  |  |  |  |  |  |
| *0 - <100 (ref)* | — | — | — | — | — | — | — | — | — | — | — | — |
| *100 - <300* |  |  |  |  | -0.28 | -0.76, 0.21 | -0.04 | -0.13, 0.05 |  |  |  |  |
| *300 - <500* |  |  |  |  | **-0.75*** | **-1.43, -0.08** | -0.12 | -0.25, 0.01 |  |  |  |  |
| *500 - 1100* |  |  |  |  | 0.26 | -0.86, 1.38 | -0.04 | -0.25, 0.18 |  |  |  |  |
| Weekly time outdoors (mins) | |  |  |  |  |  |  |  |  |  |  |  |
| *0 (ref)* | — | — | — | — | — | — | — | — | — | — | — | — |
| *>0 - <60* |  |  |  |  |  |  |  |  | **1.54***** | **0.87, 2.22** | **0.32***** | **0.18, 0.45** |
| *60 - <120* |  |  |  |  |  |  |  |  | **1.50***** | **0.79, 2.21** | **0.17*** | **0.03, 0.30** |
| *120 - <240* |  |  |  |  |  |  |  |  | **1.96***** | **1.30, 2.63** | **0.18**** | **0.05, 0.31** |
| *240 - 420* |  |  |  |  |  |  |  |  | **3.22***** | **2.63, 3.82** | **0.42***** | **0.30, 0.53** |
| Material deprivation |  |  |  |  |  |  |  |  |  |  |  |  |
| *Not in material deprivation (ref)* | | — | — | — | — | — | — | — | — | — | — | — |
| *In material deprivation* | **-5.61***** | **-7.27, -3.96** | **-1.59***** | **-1.91, -1.27** | **-6.59***** | **-7.63, -5.55** | **-1.35***** | **-1.55, -1.15** | **-7.69***** | **-8.72, -6.66** | **-1.66***** | **-1.86, -1.46** |
| **Interactions** |  |  |  |  |  |  |  |  |  |  |  |  |
| EVI x Material deprivation | |  |  |  |  |  |  |  |  |  |  |  |
| *EVI x Not in material deprivation (ref)* | | |  |  |  |  |  |  |  |  |  |  |
| *EVI x In material dep.* | -1.92 | -6.35, 2.51 | 0.75 | -0.11, 1.60 |  |  |  |  |  |  |  |  |
| Proximity to nearest GBS x Material deprivation | | |  |  |  |  |  |  |  |  |  |  |
| *<100 x Not in material deprivation (ref)* | | |  |  |  |  |  |  |  |  |  |  |
| *100 - <300 x In material deprivation* | |  |  |  | 0.46 | -0.85, 1.76 | -0.03 | -0.28, 0.22 |  |  |  |  |
| *300 - <500 x In material deprivation* | | |  |  | 0.50 | -1.36, 2.35 | 0.26 | -0.10, 0.62 |  |  |  |  |
| *500 - 1100 x In material deprivation* | | |  |  | -0.98 | -4.87, 2.91 | -0.23 | -0.98, 0.52 |  |  |  |  |
| Weekly time outdoors (mins) x Material deprivation | | |  |  |  |  |  |  |  |  |  |  |
| *0 x Not in material deprivation (ref)* | | |  |  |  |  |  |  |  |  |  |  |
| *>0 - <60 x In material deprivation* | |  |  |  |  |  |  |  | **3.19***** | **1.39, 4.99** | **0.44*** | **0.09, 0.79** |
| *60 - <120 x In material deprivation* | |  |  |  |  |  |  |  | **2.76**** | **0.70, 4.82** | **0.57**** | **0.17, 0.97** |
| *120 - <240 x In material deprivation* | |  |  |  |  |  |  |  | **2.45**** | **0.69, 4.21** | **0.88***** | **0.54, 1.22** |
| *240 - 420 x In material deprivation* | |  |  |  |  |  |  |  | **2.09**** | **0.54, 3.64** | **0.40**** | **0.10, 0.70** |
| WIMD |  |  |  |  |  |  |  |  |  |  |  |  |
| *Q1 Most deprived 20% (ref)* | | — | — | — | — | — | — | — | — | — | — | — |
| *Q2* | 0.63 | -0.06, 1.32 | 0.04 | -0.09, 0.18 | 0.62 | -0.07, 1.32 | 0.05 | -0.09, 0.18 | 0.43 | -0.26, 1.11 | 0.02 | -0.11, 0.16 |
| *Q3* | 0.57 | -0.11, 1.26 | 0.09 | -0.04, 0.22 | 0.43 | -0.27, 1.13 | 0.08 | -0.05, 0.22 | 0.29 | -0.38, 0.97 | 0.06 | -0.07, 0.19 |
| *Q4* | **1.15**** | **0.44, 1.85** | 0.03 | -0.11, 0.16 | **1.04**** | **0.31, 1.78** | 0.06 | -0.08, 0.20 | **0.86*** | **0.17, 1.55** | 0.00 | -0.14, 0.13 |
| *Q5 Least deprived 20%* | **1.37***** | **0.66, 2.08** | **0.16*** | **0.03, 0.30** | **1.01**** | **0.27, 1.75** | **0.20**** | **0.06, 0.35** | **1.02**** | **0.31, 1.72** | 0.12 | -0.01, 0.26 |
| Gender |  |  |  |  |  |  |  |  |  |  |  |  |
| *Female (ref)* | — | — | — | — | — | — | — | — | — | — | — | — |
| *Male* | **0.72***** | **0.33, 1.12** | -0.06 | -0.14, 0.01 | **0.74***** | **0.34, 1.13** | -0.06 | -0.14, 0.02 | **0.68***** | **0.29, 1.07** | -0.06 | -0.14, 0.01 |
| Age |  |  |  |  |  |  |  |  |  |  |  |  |
| *16-24 (ref)* | — | — | — | — | — | — | — | — | — | — | — | — |
| *25-44* | 0.01 | -0.67, 0.69 | -0.09 | -0.22, 0.04 | -0.01 | -0.69, 0.67 | -0.10 | -0.23, 0.03 | -0.05 | -0.73, 0.62 | -0.10 | -0.24, 0.03 |
| *45-64* | -0.11 | -0.77, 0.55 | **-0.32***** | **-0.45, -0.19** | -0.15 | -0.81, 0.51 | **-0.34***** | **-0.46, -0.21** | 0.17 | -0.49, 0.83 | **-0.28***** | **-0.40, -0.15** |
| *65-79* | **2.22***** | **1.48, 2.96** | 0.12 | -0.02, 0.27 | **2.15***** | **1.41, 2.89** | 0.11 | -0.04, 0.25 | **2.59***** | **1.86, 3.33** | **0.18*** | **0.03, 0.32** |
| *80+* | 0.81 | -0.31, 1.94 | 0.07 | -0.15, 0.28 | 0.85 | -0.28, 1.97 | 0.06 | -0.16, 0.28 | **1.75**** | **0.62, 2.89** | 0.20 | -0.02, 0.42 |
| Economic status |  |  |  |  |  |  |  |  |  |  |  |  |
| *Employed (ref)* | — | — | — | — | — | — | — | — | — | — | — | — |
| *Inactive* | **-1.28***** | **-1.79, -0.77** | **-0.24***** | **-0.34, -0.14** | **-1.27***** | **-1.78, -0.76** | **-0.25***** | **-0.34, -0.15** | **-1.09***** | **-1.59, -0.58** | **-0.21***** | **-0.31, -0.12** |
| *Unemployed* | **-1.52*** | **-2.89, -0.15** | **-0.52***** | **-0.79, -0.26** | **-1.37*** | **-2.74, -0.01** | **-0.52***** | **-0.78, -0.25** | -1.19 | -2.54, 0.17 | **-0.46***** | **-0.72, -0.19** |
| Car use |  |  |  |  |  |  |  |  |  |  |  |  |
| *No (ref)* | — | — | — | — | — | — | — | — | — | — | — | — |
| *Yes* | **1.88***** | **1.18, 2.59** | **0.33***** | **0.19, 0.46** | **1.89***** | **1.19, 2.58** | **0.33***** | **0.20, 0.47** | **1.39***** | **0.70, 2.09** | **0.27***** | **0.14, 0.41** |
| Season |  |  |  |  |  |  |  |  |  |  |  |  |
| *Autumn (ref)* | — | — | — | — | — | — | — | — | — | — | — | — |
| *Spring* | -0.05 | -0.59, 0.49 | **-0.13*** | **-0.23, -0.03** | -0.11 | -0.65, 0.44 | **-0.13*** | **-0.24, -0.03** | 0.10 | -0.44, 0.63 | **-0.11*** | **-0.21, 0.00** |
| *Summer* | 0.24 | -0.32, 0.81 | **-0.14*** | **-0.25, -0.03** | 0.21 | -0.35, 0.77 | **-0.14**** | **-0.25, -0.03** | 0.19 | -0.37, 0.74 | **-0.15**** | **-0.25, -0.04** |
| *Winter* | -0.42 | -1.01, 0.16 | -0.06 | -0.17, 0.06 | -0.47 | -1.06, 0.11 | -0.06 | -0.17, 0.05 | -0.17 | -0.75, 0.41 | -0.02 | -0.13, 0.09 |
| Wave |  |  |  |  |  |  |  |  |  |  |  |  |
| *2016-17 (ref)* | — | — | — | — | — | — | — | — | — | — | — | — |
| *2018-19* | **0.56**** | **0.15, 0.98** | 0.08 | 0.00, 0.16 | **0.70***** | **0.30, 1.11** | 0.08 | 0.00, 0.16 | **0.49*** | **0.10, 0.89** | 0.06 | -0.02, 0.13 |
| Urban status |  |  |  |  |  |  |  |  |  |  |  |  |
| *Urban (ref)* | — | — | — | — | — | — | — | — | — | — | — | — |
| *Town & fringe* | 0.53 | -0.06, 1.12 | 0.04 | -0.08, 0.15 | 0.16 | -0.48, 0.79 | -0.02 | -0.14, 0.11 | 0.28 | -0.28, 0.85 | 0.02 | -0.09, 0.13 |
| *Village, hamlet & isolated* | 0.53 | -0.03, 1.10 | 0.13* | 0.02, 0.24 | 0.12 | -0.45, 0.70 | 0.08 | -0.03, 0.19 | 0.14 | -0.35, 0.63 | **0.10*** | **0.00, 0.19** |
| Local authority |  |  |  |  |  |  |  |  |  |  |  |  |
| *Cardiff (ref)* | — | — | — | — | — | — | — | — | — | — | — | — |
| *Blaenau Gwent* |  |  |  |  | **-2.29***** | **-3.58, -0.99** | -0.04 | -0.29, 0.21 |  |  |  |  |
| *Bridgend* |  |  |  |  | -0.30 | -1.56, 0.97 | **0.27*** | **0.03, 0.51** |  |  |  |  |
| *Caerphilly* |  |  |  |  | **-2.43***** | **-3.54, -1.32** | 0.07 | -0.14, 0.29 |  |  |  |  |
| *Carmarthenshire* |  |  |  |  | **-2.42***** | **-3.57, -1.27** | -0.15 | -0.38, 0.07 |  |  |  |  |
| *Ceredigion* |  |  |  |  | -0.53 | -1.96, 0.89 | 0.25 | -0.03, 0.53 |  |  |  |  |
| *Conwy* |  |  |  |  | -0.33 | -1.59, 0.93 | 0.00 | -0.24, 0.25 |  |  |  |  |
| *Denbighshire* |  |  |  |  | -0.21 | -1.46, 1.04 | 0.10 | -0.14, 0.35 |  |  |  |  |
| *Flintshire* |  |  |  |  | -0.27 | -1.39, 0.85 | -0.02 | -0.24, 0.20 |  |  |  |  |
| *Gwynedd* |  |  |  |  | -0.45 | -1.81, 0.92 | 0.18 | -0.08, 0.45 |  |  |  |  |
| *Isle of Anglesey* |  |  |  |  | 0.13 | -1.33, 1.59 | 0.19 | -0.09, 0.48 |  |  |  |  |
| *Merthyr Tydfil* |  |  |  |  | -0.89 | -2.54, 0.75 | -0.04 | -0.36, 0.28 |  |  |  |  |
| *Monmouthshire* |  |  |  |  | **-1.29*** | **-2.44, -0.13** | 0.08 | -0.14, 0.31 |  |  |  |  |
| *Neath Port Talbot* |  |  |  |  | **-1.95**** | **-3.27, -0.64** | 0.03 | -0.22, 0.29 |  |  |  |  |
| *Newport* |  |  |  |  | **-2.22***** | **-3.34, -1.10** | -0.12 | -0.34, 0.09 |  |  |  |  |
| *Pembrokeshire* |  |  |  |  | **-1.67**** | **-2.92, -0.42** | 0.03 | -0.21, 0.27 |  |  |  |  |
| *Powys* |  |  |  |  | **-1.50**** | **-2.57, -0.43** | -0.14 | -0.35, 0.07 |  |  |  |  |
| *Rhondda Cynon Taf* |  |  |  |  | **-1.52*** | **-2.68, -0.36** | -0.10 | -0.32, 0.12 |  |  |  |  |
| *Swansea* |  |  |  |  | **-1.24*** | **-2.27, -0.21** | -0.15 | -0.35, 0.05 |  |  |  |  |
| *Torfaen* |  |  |  |  | **-3.29***** | **-4.46, -2.12** | 0.00 | -0.23, 0.23 |  |  |  |  |
| *Vale of Glamorgan* |  |  |  |  | **-1.60*** | **-2.83, -0.37** | **-0.24*** | **-0.48, -0.01** |  |  |  |  |
| *Wrexham* |  |  |  |  | **-1.50*** | **-2.79, -0.22** | -0.09 | -0.34, 0.16 |  |  |  |  |
| AIC | 56,785 |  | 31,683 |  | 56,753 |  | 31,689 |  | 56,623 |  | 31,585 |  |
| No. Obs. | 7,631 |  | 7,631 |  | 7,631 |  | 7,631 |  | 7,631 |  | 7,631 |  |
| *R^2^* | 0.097 |  | 0.100 |  | 0.106 |  | 0.105 |  | 0.117 |  | 0.113 |  |
| LRT (χ^2^)^3^ | 0.72 |  | 2.93 |  | 0.98 |  | 3.37 |  | **17.82***** | | **28.62***** | |
| ^1^**p*<0.05; ***p*<0.01; ****p*<0.001 | | | | | | | | | | | | |
| ^2^CI = Confidence Interval  ^3^LRT = Likelihood ratio tests comparing models with an interaction between the exposure and deprivation status and fully adjusted models without an interaction. P values <0.05 indicate that there is a significant difference between the models and therefore that models including an interaction term provide a better fit. | | | | | | | | | | | | |

1. **Development of residential exposure metrics**
   1. *Residential green-ness*

The residential green-ness measure is estimated as the annual mean Enhanced Vegetation Index (EVI) averaged using a 300 m buffer centred on each household location. Satellite data were used from the Landsat projects 4, 5, 7 and 8 from the USGS Earth Explorer online tool ^1^. Composite images were created from images downloaded for each year between May and July (to minimize cloud cover and coincide with peak greenness ^2^). All processing was carried out in QGIS. Data were initially processed using the Semi-Automatic Classification Plugin tool ^3^. We applied DOS1 atmospheric correction to each image as recommended by Young, et al. ^4^ and calculated EVI for each image using the vegetation index GRASS tool. Pixels covered by clouds were set to NULL by using the Cloud Masking for Landsat Products plugin to prevent these values from influencing the final green-ness density. Annual composite images of Wales were produced by mosaicking EVI images together for the same year. The 300 m buffers were clipped to the coastline to avoid underestimating greenspace available to coastal households.

- 1. *Proximity to nearest GBS*

Vector data from multiple sources were used to calculate access to GBS ^2^. These sources were: MasterMap (Topography Layer ^5^), Local green spaces ^6^); Local Government Audits; the Lle geo-portal ^7^ and OpenStreetMap (OSM) ^8^). Local authorities (LAs) are legally required to record and manage data on the open spaces that they manage through the Technical Advice Note (TAN) 16 that supplements “Planning Policy Wales”. TAN 16 data were requested from all 22 Welsh LAs regarding the open spaces they manage and 14 LAs shared their data. From Lle, we used forestry and urban tree polygon data. From OSM, park facilities such as kiosks, public toilets and road and footpath networks were extracted. Potential GBS were categorised according to a pre-defined hierarchical typology to only include blue or green spaces. These included amenities, functional spaces, seminatural habitats, enclosed and linear spaces, but excluded farmland and gardens. This typology was developed from the literature and a stakeholder workshop ^2^.

These data were combined to create a dataset of all potential GBS in Wales. The data was managed and harmonised in PostGIS. To avoid duplication when combining multiple data sources, only vectors that did not overlap with the Ordnance Survey dataset were added from TAN 16 LA data and the open data sources. We created potential access points to measure distances from each home to all GBS:

- For each regularly shaped greenspace, a proxy access point for each side was defined (e.g., North, South, East, and West)
- For linear features (e.g., rivers, canal, coastlines) an access point was defined at set intervals to reflect multiple points of access along the length of the space
- Each access point was snapped to a footpath or road network node to define a point along the network at which the GBS can be accessed

The resultant dataset was a set of point locations snapped to the road and footpath network representing access points to all GBS in Wales. This was to represent potential access because actual access points were not available nationally for all green and blue spaces. Proximity was calculated to a maximum distance of 1600 metres. It is evident from the Monitor of Engagement with the Natural Environment (MENE) data ^9,10^ that 1600m (approximately 1 mile) is the point where a rapid decline in greenspace utilisation is reported.

The distance to all GBS access points (n=3,242,803) potentially accessible within 1600m by roads and footpaths was calculated for each household in Wales and the proximity to the nearest GBS was the final measure for GBS access. The maximum proximity to nearest GBS from the home in the final analysis sample was 1,050 m.

**References**

1 USGS. *EarthExplorer*, 2020).

2 Mizen, A. *et al.* The Green, Green Grass of home: Understanding vegetation indices in relation to greenspace types at a neighbourhood level *Environment International* (Submitted).

3 QGIS Python Plugins Repository. Semi-Automatic Classification Plugin. <https://plugins.qgis.org/plugins/SemiAutomaticClassificationPlugin/>.

4 Young, N. E. *et al.* A survival guide to Landsat preprocessing. *Ecology* **98**, 920-932 (2017).

5 Ordnance Survey. *OS MasterMap Topography Layer*, 2017).

6 Natural Resources Wales. Local Green Spaces. (<https://naturalresources.wales/about-us/what-wedo/green-spaces/local-green-spaces/?lang=en>, 2020).

7 Welsh Government & Natural Resources Wales. *Lle A Geo-Portal for Wales. Access 2nd April 2020*, <<http://lle.gov.wales/home>> (2020).

8 OpenStreetMap. OSM Wales. <http://download.geofabrik.de/europe/great-britain/wales.html> (2017).

9 White, M. P., Pahl, S., Wheeler, B. W., Depledge, M. H. & Fleming, L. E. Natural environments and subjective wellbeing: Different types of exposure are associated with different aspects of wellbeing. *Health & Place* **45**, 77-84 (2017).

10 Natural England. *Monitor of Engagement with the Natural Environment (MENE)* 2021).
